# Cross-National Analysis of the Associations among Mental Disorders and Suicidal Behavior: Findings from the WHO World Mental Health Surveys

**DOI:** 10.1371/journal.pmed.1000123

**Published:** 2009-08-11

**Authors:** Matthew K. Nock, Irving Hwang, Nancy Sampson, Ronald C. Kessler, Matthias Angermeyer, Annette Beautrais, Guilherme Borges, Evelyn Bromet, Ronny Bruffaerts, Giovanni de Girolamo, Ron de Graaf, Silvia Florescu, Oye Gureje, Josep Maria Haro, Chiyi Hu, Yueqin Huang, Elie G. Karam, Norito Kawakami, Viviane Kovess, Daphna Levinson, Jose Posada-Villa, Rajesh Sagar, Toma Tomov, Maria Carmen Viana, David R. Williams

**Affiliations:** 1 Harvard University, Department of Psychology, Cambridge, Massachusetts, United States of America; 2 Department of Health Care Policy, Harvard Medical School, Boston, Massachusetts, United States of America; 3 Center for Public Mental Health, Gösing am Wagram, Austria; 4 University of Otago, Christchurch, New Zealand; 5 Department of Epidemiological Research, Division of Epidemiological and Psychosocial Research, National Institute of Psychiatry (Mexico) & Metropolitan Autonomous University, Mexico City, Mexico; 6 Department of Psychiatry, State University of New York at Stony Brook, Stony Brook, New York, United States of America; 7 University Hospital Gasthuisberg, Leuven, Belgium; 8 IRCCS Centro S. Giovanni di Dio Fatebenefratelli, Brescia, Italy; 9 Netherlands Institute of Mental Health and Addiction, Utrecht, The Netherlands; 10 Public Health Research and Evidence Based Medicine Department, National School of Public Health and Health Services Management, Bucharest, Romania; 11 University College Hospital, Ibadan, Nigeria; 12 Sant Joan de Deu-SSM, Barcelona, Ciber en Salud Mental (CIBERSAM), ISCIII, Barcelona, Spain; 13 Shenzhen Institute of Mental Health & Shenzhen Kangning Hospital, Shenzhen, People's Republic of China; 14 Institute of Mental Health, Peking University, Beijing, People's Republic of China; 15 Department of Psychiatry & Clinical Psychology, St. George Hospital University Medical Center, Balamand University, Faculty of Medicine and the Institute for Development, Research, Advocacy & Applied Care (IDRAAC), Medical Institute for Neuropsychological Disorders (MIND), Beirut, Lebanon; 16 Department of Mental Health, School of Public Health, University of Tokyo, Tokyo, Japan; 17 University of Paris Descartes, EA 4069, MGEN Foundation for Public Health, Paris, France; 18 Research & Planning, Mental Health Services Ministry of Health, Jerusalem, Israel; 19 Pontificia Universidad Javeriana, Centro Medico de la Sabana, Bogota, Colombia; 20 Department of Psychiatry, All India Institute of Medical Sciences, New Delhi, India; 21 New Bulgarian University, Institute for Human Relations, Sofia, Bulgaria; 22 Section of Psychiatric Epidemiology, Institute of Psychiatry, School of Medicine, University of São Paulo, São Paulo, Brazil; 23 Department of Society, Human Development, and Health, Harvard School of Public Health, Boston, Massachusetts, United States of America; King's College London, United Kingdom

## Abstract

**Background:**

Suicide is a leading cause of death worldwide. Mental disorders are among the strongest predictors of suicide; however, little is known about which disorders are uniquely predictive of suicidal behavior, the extent to which disorders predict suicide attempts beyond their association with suicidal thoughts, and whether these associations are similar across developed and developing countries. This study was designed to test each of these questions with a focus on nonfatal suicide attempts.

**Methods and Findings:**

Data on the lifetime presence and age-of-onset of Diagnostic and Statistical Manual of Mental Disorders, 4th Edition (DSM-IV) mental disorders and nonfatal suicidal behaviors were collected via structured face-to-face interviews with 108,664 respondents from 21 countries participating in the WHO World Mental Health Surveys. The results show that each lifetime disorder examined significantly predicts the subsequent first onset of suicide attempt (odds ratios [ORs] = 2.9–8.9). After controlling for comorbidity, these associations decreased substantially (ORs = 1.5–5.6) but remained significant in most cases. Overall, mental disorders were equally predictive in developed and developing countries, with a key difference being that the strongest predictors of suicide attempts in developed countries were mood disorders, whereas in developing countries impulse-control, substance use, and post-traumatic stress disorders were most predictive. Disaggregation of the associations between mental disorders and nonfatal suicide attempts showed that these associations are largely due to disorders predicting the onset of suicidal thoughts rather than predicting progression from thoughts to attempts. In the few instances where mental disorders predicted the transition from suicidal thoughts to attempts, the significant disorders are characterized by anxiety and poor impulse-control. The limitations of this study include the use of retrospective self-reports of lifetime occurrence and age-of-onset of mental disorders and suicidal behaviors, as well as the narrow focus on mental disorders as predictors of nonfatal suicidal behaviors, each of which must be addressed in future studies.

**Conclusions:**

This study found that a wide range of mental disorders increased the odds of experiencing suicide ideation. However, after controlling for psychiatric comorbidity, only disorders characterized by anxiety and poor impulse-control predict which people with suicide ideation act on such thoughts. These findings provide a more fine-grained understanding of the associations between mental disorders and subsequent suicidal behavior than previously available and indicate that mental disorders predict suicidal behaviors similarly in both developed and developing countries. Future research is needed to delineate the mechanisms through which people come to think about suicide and subsequently progress from ideation to attempts.

*Please see later in the article for Editors' Summary*

## Abstract 2

Using data from over 100,000 individuals in 21 countries participating in the WHO World Mental Health Surveys, Matthew Nock and colleagues investigate which mental health disorders increase the odds of experiencing suicidal thoughts and actual suicide attempts, and how these relationships differ across developed and developing countries.

## Editors' Summary

**Background:**

Suicide is a leading cause of death worldwide. Every 40 seconds, someone somewhere commits suicide. Over a year, this adds up to about 1 million self-inflicted deaths. In the USA, for example, where suicide is the 11th leading cause of death, more than 30,000 people commit suicide every year. The figures for nonfatal suicidal behavior (suicidal thoughts or ideation, suicide planning, and suicide attempts) are even more shocking. Globally, suicide attempts, for example, are estimated to be 20 times as frequent as completed suicides. Risk factors for nonfatal suicidal behaviors and for suicide include depression and other mental disorders, alcohol or drug abuse, stressful life events, a family history of suicide, and having a friend or relative commit suicide. Importantly, nonfatal suicidal behaviors are powerful predictors of subsequent suicide deaths so individuals who talk about killing themselves must always be taken seriously and given as much help as possible by friends, relatives, and mental-health professionals.

**Why Was This Study Done?:**

Experts believe that it might be possible to find ways to decrease suicide rates by answering three questions. First, which individual mental disorders are predictive of nonfatal suicidal behaviors? Although previous studies have reported that virtually all mental disorders are associated with an increased risk of suicidal behaviors, people often have two or more mental disorders (“comorbidity”), so many of these associations may reflect the effects of only a few disorders. Second, do some mental disorders predict suicidal ideation whereas others predict who will act on these thoughts? Finally, are the associations between mental disorders and suicidal behavior similar in developed countries (where most studies have been done) and in developing countries? By answering these questions, it should be possible to improve the screening, clinical risk assessment, and treatment of suicide around the world. Thus, in this study, the researchers undertake a cross-national analysis of the associations among mental disorders (as defined by the Diagnostic and Statistical Manual of Mental Disorders, 4th Edition [DSM-IV]) and nonfatal suicidal behaviors.

**What Did the Researchers Do and Find?:**

The researchers collected and analyzed data on the lifetime presence and age-of-onset of mental disorders and of nonfatal suicidal behaviors in structured interviews with nearly 110,000 participants from 21 countries (part of the World Health Organization's World Mental Health Survey Initiative). The lifetime presence of each of the 16 disorders considered (mood disorders such as depression; anxiety disorders such as post-traumatic stress disorder [PTSD]; impulse-control disorders such as attention deficit/hyperactivity disorder; and substance misuse) predicted first suicide attempts in both developed and developing countries. However, the increased risk of a suicide attempt associated with each disorder varied. So, for example, in developed countries, after controlling for comorbid mental disorders, major depression increased the risk of a suicide attempt 3-fold but drug abuse/dependency increased the risk only 2-fold. Similarly, although the strongest predictors of suicide attempts in developed countries were mood disorders, in developing countries the strongest predictors were impulse-control disorders, substance misuse disorders, and PTSD. Other analyses indicate that mental disorders were generally more predictive of the onset of suicidal thoughts than of suicide plans and attempts, but that anxiety and poor impulse-control disorders were the strongest predictors of suicide attempts in both developed and developing countries.

**What Do These Findings Mean?:**

Although this study has several limitations—for example, it relies on retrospective self-reports by study participants—its findings nevertheless provide a more detailed understanding of the associations between mental disorders and subsequent suicidal behaviors than previously available. In particular, its findings reveal that a wide range of individual mental disorders increase the chances of an individual thinking about suicide in both developed and developing countries and provide new information about the mental disorders that predict which people with suicidal ideas will act on such thoughts. However, the findings also show that only half of people who have seriously considered killing themselves have a mental disorder. Thus although future suicide prevention efforts should include a focus on screening and treating mental disorders, ways must also be found to identify the many people without mental disorders who are at risk of suicidal behaviors.

**Additional Information:**

Please access these Web sites via the online version of this summary at http://dx.doi.org/10.1371/journal.pmed.1000123.

- The US National Institute of Mental Health provides information about suicide in the US: statistics and prevention
- The UK National Health Service provides information about suicide, including statistics about suicide in the UK and links to other resources
- The World Health Organization provides global statistics about suicide and information on suicide prevention
- MedlinePlus provides links to further information and advice about suicide and about mental health (in English and Spanish)
- Further details about the World Mental Health Survey Initiative and about DSM-IV are available

## Introduction

Suicide is among the leading causes of death and disease burden around the world [1]–[4]. Although there have been significant advances in suicide research as well as increases in the treatment of suicidal people, the rate of suicidal behaviors has not changed as a result [1],[5]. The seriousness of this problem has led the World Health Organization (WHO) [6], the U.S. Surgeon General [7], the U.S. Department of Health and Human Services [8], and the Institute of Medicine [2] to call for research aimed at better understanding the risk factors for suicide and nonfatal suicidal behavior. Nonfatal suicidal behaviors are important because they are among the most powerful predictors of subsequent suicide deaths [9]–[12] and because they are significant outcomes in their own right that cause substantial distress.

Most research aimed at identifying risk factors for nonfatal suicidal behaviors has focused on the importance of prior mental disorders. To date, dozens of studies have clearly shown that mental disorders, particularly depressive disorders, are among the strongest risk factors for suicide attempts and suicide deaths [12]–[14]. Despite this knowledge, several important questions remain regarding how and why mental disorders are associated with suicidal behavior. First, little is known about which disorders are *uniquely* predictive of suicidal behavior. Most studies have examined the bivariate associations between individual disorders and suicidal behavior [15]–[18] and in doing so have shown that virtually all mental disorders are associated with an increased risk of suicidal behavior [5],[12],[19]. However, because mental disorders are highly comorbid [20], it is possible that many of the observed associations are due to the true effects of only a small number of disorders. For instance, several studies have reported that panic disorder is a significant risk factor for suicide attempts [15],[21]. Other studies, though, have shown that this association is no longer significant after accounting for a broad range of comorbid disorders [22]–[24]. Obtaining a clearer picture regarding which disorders are uniquely related to suicidal behavior is necessary in order to better understand, predict, and treat suicidal behavior and requires that future studies more carefully examine and control for the effects of comorbidity.

Second, surprisingly little is known about the extent to which mental disorders predict suicide attempts beyond their association with suicide ideation (i.e., suicidal thoughts). Several recent studies have suggested that although mental disorders are strongly predictive of suicide ideation, they are less useful in predicting which people with suicide ideation go on to make suicide plans and attempts [25],[26]. If this initial finding is replicated more broadly, an important next question is whether some disorders are more useful in predicting suicide ideation, while others are more predictive of acting on such thoughts. Providing greater clarity regarding how different mental disorders relate to distinct stages of the progression to suicide attempts would not only advance knowledge regarding how suicidal behavior develops, but would provide valuable information for clinical risk assessment and treatment.

Third, there is debate regarding the generality of prior findings on the association between mental disorders and suicidal behavior. Previous studies have been conducted almost exclusively in developed countries [27]–[29], and several recent studies in developing countries have suggested that mental disorders are less predictive of suicidal behaviors in such countries [30],[31]. Although, one recent study reported no difference between developed and developing countries when similar assessment methods are used cross-nationally [19]. Accurate data on the risk factors for suicidal behavior in both developed and developing countries are needed to develop more adequate screening, prevention, and intervention programs around the world [2],[3],[30].

The current study was designed to address each of these limitations by conducting a comprehensive cross-national analysis of the associations between lifetime history and age-of-onset of mental disorders and the subsequent first onset of nonfatal suicidal behaviors using data from the WHO World Mental Health (WMH) Surveys—a series of coordinated epidemiological surveys carried out in 21 countries around the world [32],[33]. The objectives of this study were to examine the unique associations between prior mental disorders and the subsequent first onset of suicidal behaviors, to test the effects of comorbidity on suicidal behaviors, and to decompose the associations between mental disorders and suicidal behaviors by considering effects of mental disorders on suicide ideation, plans among ideators, and suicide attempts separately among ideators with and without suicide plans. This study extends the work of prior studies that have reported on the bivariate associations between mental disorders and suicidal behaviors in a subset of WMH countries [19] and that have tested multivariate models of these associations within individual WMH countries [26]. The size and representativeness of the current study sample provided a unique opportunity to test complex multivariate models predicting the onset of suicidal behaviors and to examine the consistency of the observed effects between developed and developing countries.

## Methods

### Respondent Samples

The WMH surveys were carried out in 21 countries in: Africa (Nigeria; South Africa); the Americas (Brazil; Colombia; Mexico; United States), Asia and the Pacific (India; Japan; New Zealand; Beijing, Shanghai, and Shenzhen in the Peoples Republic of China), Europe (Belgium; Bulgaria; France; Germany; Italy; the Netherlands; Romania; Spain; Ukraine); and the Middle East (Israel; Lebanon). The World Bank [34] classifies Brazil, Bulgaria, China, Colombia, India, Lebanon, Mexico, Nigeria, Romania, South Africa, and Ukraine as low- and lower-middle income countries (i.e., developing); and all other survey countries as higher-middle or high-income countries (i.e., developed). Respondents were selected in most WMH countries using a stratified multistage clustered-area probability sampling strategy. The total sample size was 108,664, with individual country sample sizes ranging from 2,357 in Romania to 12,790 in New Zealand. The weighted average response rate across all countries was 73.3% (see Table 1 for sample and survey characteristics).

**Table 1:** WMH sample characteristics—developed and developing countries.

Country	Survey^a^	Sample Characteristics^b^	Field Dates	Age Range (y)	*n* (Part 1)	*n* (Part II)	*n* (Part II and Age≤44 y^c^)	Response Rate^d^^,^ e
I. Developed								
Belgium	ESEMeD	Stratified multistage clustered probability sample of individuals residing in households from the national register of Belgium residents. NR	2001–2002	18+	2,419	1,043	486	50.6
France	ESEMeD	Stratified multistage clustered sample of working telephone numbers merged with a reverse directory (for listed numbers). Initial recruitment was by telephone, with supplemental in-person recruitment in households with listed numbers. NR	2001–2002	18+	2,894	1,436	727	45.9
Germany	ESEMeD	Stratified multistage clustered probability sample of individuals from community resident registries. NR	2002–2003	18+	3,555	1,323	621	57.8
Israel	NHS	Stratified multistage clustered area probability sample of individuals from a national resident register. NR	2002–2004	21+	4,859	—	—	72.6
Italy	ESEMeD	Stratified multistage clustered probability sample of individuals from municipality resident registries. NR	2001–2002	18+	4,712	1,779	853	71.3
Japan	WMHJ2002–2006	Unclustered two-stage probability sample of individuals residing in households in ten metropolitan areas (Fukiage, Higashi-ichiki, Ichiki, Kushikino, Nagasaki, Okayama, Sano, Tamano, Tendo, and Tochigi)	2002–2006	20+	3,416	1,305	425	59.2
Netherlands	ESEMeD	Stratified multistage clustered probability sample of individuals residing in households that are listed in municipal postal registries. NR	2002–2003	18+	2,372	1,094	516	56.4
New Zealand	NZMHS	Stratified multistage clustered area probability sample of household residents. NR	2004–2005	18+	12,790	7,312	—	73.3
Spain	ESEMeD	Stratified multistage clustered area probability sample of household residents. NR	2001–2002	18+	5,473	2,121	960	78.6
United States	NCS-R	Stratified multistage clustered area probability sample of household residents. NR	2002–2003	18+	9,281	5,691	3,196	70.9
II. Developing								
Brazil	São Paulo Megacity	Stratified multistage clustered area probability sample of household residents in the São Paulo metropolitan area.	2005–2007	18+	5,037	2,942	—	81.3
Bulgaria	NSHS	Stratified multistage clustered area probability sample of household residents. NR	2003–2007	18+	5,318	2,233	741	72.0
Colombia	NSMH	Stratified multistage clustered area probability sample of household residents in all urban areas of the country (approximately 73% of the total national population)	2003	18–65	4,426	2,381	1,731	87.7
India	WMHI	Stratified multistage clustered area probability sample of household residents in Pondicherry region. NR	2003–2005	18+	2,992	1,373	825	98.6
Lebanon	LEBANON	Stratified multistage clustered area probability sample of household residents. NR	2002–2003	18+	2,857	1,031	595	70.0
Mexico	M-NCS	Stratified multistage clustered area probability sample of household residents in all urban areas of the country (approximately 75% of the total national population).	2001–2002	18–65	5,782	2,362	1,736	76.6
Nigeria	NSMHW	Stratified multistage clustered area probability sample of households in 21 of the 36 states in the country, representing 57% of the national population. The surveys were conducted in Yoruba, Igbo, Hausa, and Efik languages.	2002–2003	18+	6,752	2,143	1,392	79.3
PRC	B-WMH S-WMH	Stratified multistage clustered area probability sample of household residents in the Beijing and Shanghai metropolitan areas.	2002–2003	18+	5,201	1,628	859	74.7
PRC	Shenzhen	Stratified multistage clustered area probability sample of household residents and temporary residents in the Shenzhen area.	2006–2007	18+	7,132	2,475	2,190	80.0
Romania	RMHS	Stratified multistage clustered area probability sample of household residents. NR	2005–2006	18+	2,357	—	—	70.9
South Africa	SASH	Stratified multistage clustered area probability sample of household residents. NR	2003–2004	18+	4,315	—	—	87.1
Ukraine	CMDPSD	Stratified multistage clustered area probability sample of household residents. NR	2002	18+	4,724	1,719	714	78.3

a B-WMH, The Beijing World Mental Health Survey; CMDPSD, Comorbid Mental Disorders during Periods of Social Disruption; ESEMeD, The European Study Of The Epidemiology Of Mental Disorders; LEBANON, Lebanese Evaluation of the Burden of Ailments and Needs of the Nation; M-NCS, The Mexico National Comorbidity Survey; NCS-R, The US National Comorbidity Survey Replication; NHS, Israel National Health Survey; NSHS, Bulgaria National Survey of Health and Stress; NSMH, The Colombian National Study of Mental Health; NSMHW, The Nigerian Survey of Mental Health and Wellbeing; NZMHS, New Zealand Mental Health Survey; RMHS, Romania Mental Health Survey; SASH, South Africa Stress and Health Study; S-WMH, The Shanghai World Mental Health Survey; WMHI, World Mental Health India; WMHJ2002–2006, World Mental Health Japan Survey.

b Most WMH surveys are based on stratified multistage clustered area probability household samples in which samples of areas equivalent to counties or municipalities in the US were selected in the first stage followed by one or more subsequent stages of geographic sampling (e.g., towns within counties, blocks within towns, households within blocks) to arrive at a sample of households, in each of which a listing of household members was created and one or two people were selected from this listing to be interviewed. No substitution was allowed when the originally sampled household resident could not be interviewed. These household samples were selected from census area data in all countries other than France (where telephone directories were used to select households) and the Netherlands (where postal registries were used to select households). Several WMH surveys (Belgium, Germany, Italy) used municipal resident registries to select respondents without listing households. The Japanese sample is the only totally unclustered sample, with households randomly selected in each of the four sample areas and one random respondent selected in each sample household. 16 of the 22 surveys are based on nationally representative (NR) household samples, while two others are based on NR household samples in urbanized areas (Colombia, Mexico).

c Brazil, Israel, New Zealand, Romania, and South Africa did not have an age restricted part II sample. All other countries, with the exception of India, Nigeria, the People's Republic of China, and Ukraine (which were age restricted to ≤39 y) were age restricted to ≤44 y.

d The response rate is calculated as the ratio of the number of households in which an interview was completed to the number of households originally sampled, excluding from the denominator households known not to be eligible either because of being vacant at the time of initial contact or because the residents were unable to speak the designated languages of the survey.

e The weighted average response rate is 73%.

### Procedures

All surveys were conducted face-to-face by trained lay interviewers. Standardized interviewer training procedures, WHO translation protocols for all study materials, and quality control procedures for interviewer and data accuracy, which have been consistently employed across all WMH countries, are described in more detail elsewhere [33],[35] [36]. All respondents completed a part I interview that contained assessments of the lifetime prevalence and age-of-onset of core mental disorders as well as the assessment of the lifetime prevalence and ages-of-onset of three nonfatal suicidal behaviors—suicidal ideation, suicide plans, and suicide attempts. All part I respondents who met criteria for any disorder and a probability subsample of approximately 25% of the rest of the respondents were administered a part II interview that assessed potential correlates and disorders of secondary interest (*n* = 54,992). The part II data, which are used in the current report, were weighted to adjust for this differential sampling of part I respondents into part II, for differential probabilities of selection within households, and for discrepancies between sample and Census population distributions of socio-demographic variables. These weighting procedures make the part II sample representative of the entire population despite the undersampling of part I respondents who did not have any core Diagnostic and Statistical Manual of Mental Disorders, 4th Edition (DSM-IV) disorder. Informed consent was obtained before beginning interviews in all countries. Procedures for obtaining informed consent and protecting human subjects were approved and monitored for compliance by the Institutional Review Boards of organizations coordinating surveys in each country.

### Measures

#### DSM-IV mental disorders

Lifetime mental disorders were assessed using the WHO Composite International Diagnostic Interview (CIDI) Version 3.0, a fully structured diagnostic interview administered by trained lay interviewers [33]. The 16 mental disorders assessed in the CIDI were anxiety disorders (panic, generalized anxiety, phobias, posttraumatic stress [PTSD], and separation anxiety), mood disorders (major depressive, dysthymic, and bipolar), impulse-control disorders (oppositional-defiant, conduct, attention-deficit/hyperactivity, and intermittent explosive), and substance use disorders (alcohol and illicit drug abuse and dependence). Diagnoses were made using organic exclusion rules but without using diagnostic hierarchy rules, leading to an inflation in the estimates of comorbidity compared to those that would have been made if hierarchy rules had been used. Clinical reappraisal studies found CIDI diagnoses to have generally good concordance with blinded clinical diagnoses based on the Structured Clinical Interview for DSM-IV [37] in probability subsamples of respondents from the France, Italy, Spain, and US surveys [38],[39]. Concordance in the assessment of comorbidity, though, was not evaluated in these studies, nor were CIDI clinical reappraisal studies carried out in any of the developing countries included in the WMH series.

#### Suicidal behavior

Lifetime nonfatal suicidal behaviors were assessed using the Suicidality Module of the CIDI [33], which includes assessments of the occurrence and age-of-onset of suicide ideation, plans, and attempts. This assessment was included in the part I sample, however, as several of the mental disorders were assessed in part II of the CIDI, all multivariate analyses were restricted to part II cases. As noted above, though, the weighted part II sample is representative of the population and includes 100% of respondents with core DSM-IV disorders, which means that there is no loss in generality of results due to the focus on part II respondents. Consistent with our goal of examining relationships of mental disorders with a continuum of suicidal behaviors, we considered five dated lifetime outcomes in a series of nested survival analyses (see below for analysis methods): (1) first suicide attempt in the total sample; (2) first onset of suicide ideation in the total sample; (3) first suicide plan among respondents with a lifetime history of ideation; (4) first suicide attempt among respondents with a history of a lifetime suicide plan; and (5) first unplanned suicide attempt among respondents with a history of ideation.

### Analysis Methods

We first estimated the prevalence of each temporally prior mental disorder among respondents with each of the five suicidal outcomes using cross-tabulations. The temporal priority of mental disorders was examined using individual-level retrospective age-of-onset reports for both mental disorders and suicidal behaviors. Next, the associations among temporally prior mental disorders (i.e., time-varying covariates) and subsequent suicidal behaviors were estimated using discrete-time survival models with person-year as the unit of analysis [40]. In this approach, each year of life of each respondent up to and including the year of first onset of the outcome is treated as a separate observational record, with years prior to the onset coded 0 on the outcome variable and the year of onset coded 1 on the outcome variable. All years subsequent to age-of-onset of the outcome are excluded from the analysis. All years of life up to the age at interview are included for respondents who never experienced the outcome. Each person-year record is coded for the respondent's age in that year, age at interview, sex, and prior history of each mental disorder as of that age. This data array is analyzed using logistic regression methods, with dummy predictor variables for age in the person-year used as control variables along with predictors for age at interview, sex, and lifetime history of mental disorders as of the person-year in predicting first onset of the outcome. The logistic regression coefficients can be interpreted as survival coefficients because of the exclusion from the data array of person-years after the onset of the outcome.

We used this approach to estimate discrete-time survival models that were bivariate (i.e., including only one disorder at a time) as well as models that were multivariate (i.e., including all disorders simultaneously) in predicting each suicidal behavior. These models all controlled for respondent age at interview (both linear and quadratic terms in addition to a series of dummy variables for broad age cohort groups of respondents in the age ranges 18–29, 30–44, 45–59, and 60+ y at the time of interview), age in each person-year of the survival data file, and sex. Results regarding the associations between these control variables and the outcomes have been reported previously [19] and will not be repeated here. We also estimated a series of models that allowed for multiplicative interactions among comorbid disorders. Because 16 different mental disorders were included as predictors in the analyses, the number of possible interactions was substantial (2^16^−17 = 65,519), and so we imposed some structure on the interactions to generate models with stable estimates.

We began with a model with summary dummy variables for the total number of disorders experienced by each respondent (e.g., separate dummy predictor variables to distinguish respondents with exactly two disorders, exactly three, …, etc.). This model assumed that interactions were constant across type of disorder and were influenced only by number of disorders (i.e., that the 120 odds ratios [ORs] of the 16×15/2 logically possible two-way interactions could all be considered the same; that the 560 ORs of the 16×15×14/6 logically possible three-way interactions could all be considered the same, etc.). Next, more complex models were estimated allowing for interactions between each type of disorder and number of comorbid disorders. The simple model assuming constant interactions was a good approximation of the data, so that model was used in subsequent analyses. Finally, population attributable risk proportions (PARPs) due to each class of disorders were calculated on the basis of the results of the simple interaction model.

Survival coefficients were exponentiated to generate ORs and their standard errors for ease of interpretation. Standard errors of prevalence estimates and survival coefficients were estimated with the Taylor series method [41] using SUDAAN software [42] to adjust for weighting and clustering. Multivariate significance was evaluated with Wald χ^2^ tests based on design-corrected coefficient variance–covariance matrices. All significance tests were evaluated using 0.05-level two-sided tests. All analyses were conducted two times—one time each for developed and developing countries—in order to test the consistency of the observed effects.

## Results

### Prevalence of Temporally Prior Mental Disorders among those with Suicidal Behavior

Approximately half of people who had seriously considered killing themselves reported a prior lifetime DSM-IV disorder in both developed (51.8%) and developing (42.9%) countries (χ^2^_1_ = 16.9, *p*<0.001). Mental disorders were even more prevalent among respondents who went on to make a suicide attempt (65.7% and 54.6%, respectively; χ^2^_1_ = 15.4, *p*<0.001). Among respondents with suicide ideation, the prevalence of a mental disorder was higher among those making a planned suicide attempt (68.6% and 59.6%; χ^2^_1_ = 6.7, *p* = 0.009) than among those making an unplanned (i.e., impulsive) attempt (60.5% and 44.6%; χ^2^_1_ = 12.6, *p*<0.001). The rates of mental disorders were higher among those making a planned attempt than an unplanned attempt in both developed (χ^2^_1_ = 5.4, *p* = 0.020) and developing (χ^2^_1_ = 15.9, *p*<0.001) countries. This rate suggests that impulsive attempts may be more influenced by other factors such as stressful life events (see Tables S1 and S2 for more detailed results).

### Associations of Temporally Primary DSM-IV Disorders with Suicide Attempts

Bivariate survival models reveal that each of the 16 DSM-IV disorders examined is significantly associated with the onset of a subsequent suicide attempt, with ORs ranging from 2.9 (specific phobia) to 7.1 (bipolar disorder) (interquartile range [IQR] = 3.7–5.5) in developed countries and from 2.6 (agoraphobia) to 8.9 (conduct disorder) (IQR = 3.7–6.9) in developing countries (Tables 2 and 3). These ORs all decreased in additive multivariate models that test the unique associations between disorders and suicide attempt in both developed countries, where ORs range from 0.9 (dysthymia) to 2.9 (major depression) (IQR = 1.3–2.0), and in developing countries, where ORs range from 1.3 (agoraphobia and OCD) to 3.3 (PTSD) (IQR = 1.4–2.2)—although 30 of the 32 ORs remained greater than 1.0, and 18 remain statistically significant. Next, simple interactive multivariate models were estimated that included one dummy variable for each of the 16 disorders plus additional dummy variables for each number of disorders (e.g., exactly two prior disorders, exactly three, etc.). The ORs for individual disorders in these models can be interpreted as the relative odds of a subsequent suicide attempt among respondents with a pure disorder (i.e., only this one disorder) versus those with no disorders. Similar to the additive multivariate model, pure disorders have consistently significant ORs that are generally comparable in magnitude in developed countries, where ORs range from 1.5 (dysthymia) to 3.5 (bipolar disorder) (IQR = 1.9–2.6), and in developing countries, where ORs range from 2.1 (agoraphobia) to 5.6 (PTSD) (IQR = 2.7–3.7). In developed countries, the disorders with the strongest association with suicide attempts in addition to bipolar disorder were depression (OR = 3.2), and PTSD (OR = 3.0), whereas in developing countries, the strongest predictors in addition to PTSD were conduct disorder (OR = 4.8), and drug abuse/dependence (OR = 4.0).

**Table 2:** Survival models of the associations between DSM-IV disorders and a subsequent suicide attempt—developed countries.

	Response Variable: Lifetime Attempt among Part II Sample (*n* = 27,963)		
	Bivariate Models^a^	Multivariate Additive Model^b^	Multivariate Interactive Model^c^
DSM-IV Disorders	OR (95% CI)	OR (95% CI)	OR (95% CI)
I. Anxiety disorders			
Panic disorder	5.1 (4.0–6.4)*	1.5 (1.1–2.1)*	2.3 (1.6–3.3)*
GAD	5.6 (4.6–6.7)*	1.8 (1.4–2.3)*	2.5 (1.9–3.4)*
Specific phobia	2.9 (2.5–3.4)*	1.6 (1.4–1.9)*	1.9 (1.5–2.4)*
Social phobia	3.8 (3.2–4.4)*	1.5 (1.2–1.8)*	1.9 (1.4–2.5)*
PTSD	6.5 (5.4–7.6)*	2.1 (1.7–2.7)*	3.0 (2.3–3.8)*
SAD	3.5 (2.8–4.4)*	1.0 (0.8–1.3)	1.6 (1.2–2.1)*
Agoraphobia	3.7 (2.7–5.0)*	1.4 (0.9–2.1)	2.2 (1.4–3.4)*
Any anxiety disorder	4.6 (4.0–5.3)*	—	—
II. Mood disorders			
MDD	5.8 (5.0–6.8)*	2.9 (2.4–3.6)*	3.2 (2.5–4.2)*
Dysthymia	5.1 (4.0–6.6)*	0.9 (0.6–1.3)	1.5 (1.0–2.2)*
Bipolar disorder	7.1 (5.5–9.3)*	2.3 (1.6–3.3)*	3.5 (2.4–5.3)*
Any Mood disorder	5.9 (5.1–6.8)*	—	—
III. Impulse-control disorders			
ODD	5.3 (4.1–6.7)*	1.6 (1.1–2.2)*	2.2 (1.6–3.0)*
Conduct disorder	4.8 (3.6–6.4)*	1.5 (1.1–2.1)*	2.1 (1.4–3.0)*
ADHD	4.6 (3.5–6.1)*	1.4 (1.0–1.9)	1.9 (1.3–2.7)*
IED	3.3 (2.5–4.3)*	1.2 (0.8–1.7)	1.8 (1.2–2.5)*
Any impulse-control disorder	4.3 (3.5–5.3)*	—	—
IV. Substance use disorders			
Alcohol abuse or dependency	4.4 (3.7–5.3)*	2.1 (1.6–2.6)*	2.6 (1.9–3.5)*
Drug abuse or dependency	4.9 (3.8–6.2)*	1.3 (0.9–1.8)	2.0 (1.4–2.8)*
Any substance use disorder	4.8 (4.0–5.8)*	—	
V. Any disorder	6.4 (5.5–7.5)*	—	
Overall group effect χ^2^ (*p*-value)^d^		1,151.6 (<0.001)*	160.5 (<0.001)*

Empty cells indicate the disorder specified in the row was not included in the model.

a Bivariate models (each disorder in a separate discrete time survival model) include the following controls: age, age-squared, age cohorts, sex, and person-year.

b Multivariate additive model (all disorders together in a discrete time survival model) includes the following covariates: age, age-squared, age cohorts, sex, and person-year.

c Multivariate interactive model (all disorders together in a discrete time survival model controlling for number of disorders as interactions) includes the following covariates: age, age-squared, age cohorts, sex, and person-year.

d The group effect χ^2^ tests the set of coefficients for type of disorder for significance, while the individual χ^2^ only test presence versus absence of each DSM-IV/CIDI disorder.

*: Significant at the 0.05 level, two-sided test.

**Abbreviations:** ADHD, Attention Deficit Hyperactivity Disorder; CI, confidence interval; GAD, Generalized Anxiety Disorder; IED, Intermittent Explosive Disorder; MDD, Major Depressive Disorder; OCD, Obsessive Compulsive Disorder; ODD, Oppositional Defiant Disorder; SAD, Separation Anxiety Disorder.

**Table 3:** Survival models of the associations between DSM-IV disorders and a subsequent suicide attempt—developing countries.

	Response Variable: Lifetime Attempt among Part II Sample (*n* = 26,959)		
	Bivariate Models^a^	Multivariate Additive Model^b^	Multivariate Interactive Model^c^
DSM-IV Disorders	OR (95% CI)	OR (95% CI)	OR (95% CI)
I. Anxiety disorders			
Panic disorder	5.0 (3.4–7.3)*	1.6 (0.9–2.9)	3.0 (1.7–5.4)*
GAD	5.4 (3.9–7.5)*	1.4 (0.9–2.2)	2.9 (1.8–4.6)*
Specific phobia	3.3 (2.8–4.0)*	1.9 (1.5–2.3)*	2.6 (2.0–3.4)*
Social phobia	3.9 (3.0–5.0)*	1.7 (1.2–2.4)*	2.9 (2.0–4.0)*
PTSD	8.3 (6.0–11.6)*	3.3 (2.1–5.1)*	5.6 (3.5–8.8)*
OCD	3.4 (2.0–6.1)*	1.3 (0.7–2.4)	2.3 (1.3–4.2)*
SAD	2.9 (2.1–4.0)*	1.4 (1.0–1.9)	2.4 (1.7–3.4)*
Agoraphobia	2.6 (1.8–3.7)*	1.3 (0.9–1.8)	2.1 (1.4–3.0)*
Any anxiety disorder	3.7 (3.2–4.4)*	—	—
II. Mood disorders			
MDD	5.1 (4.2–6.1)*	2.2 (1.7–2.9)*	3.2 (2.4–4.3)*
Dysthymia	7.1 (4.8–10.7)*	1.8 (1.0–3.4)	3.5 (1.9–6.6)*
Bipolar disorder	6.7 (4.4–10.0)*	1.4 (0.8–2.4)	3.2 (1.8–5.4)*
Any mood disorder	5.0 (4.2–6.0)*	—	—
III. Impulse-control disorders			
ODD	6.7 (4.1–10.9)*	1.9 (1.0–3.6)	3.6 (2.0–6.5)*
Conduct disorder	8.9 (5.6–14.2)*	2.4 (1.2–4.9)*	4.8 (2.5–9.1)*
ADHD	4.9 (3.2–7.7)*	1.5 (0.8–2.8)	2.8 (1.6–4.9)*
IED	4.7 (3.7–6.2)*	2.0 (1.4–2.9)*	3.3 (2.3–4.7)*
Any Impulse-control disorder	5.4 (4.3–6.9)*	—	—
IV. Substance use disorders			
Alcohol abuse or dependency	4.8 (3.7–6.1)*	2.5 (1.8–3.6)*	3.7 (2.6–5.4)*
Drug abuse or dependency	7.5 (5.4–10.4)*	2.1 (1.2–3.6)*	4.0 (2.5–6.4)*
Any substance use disorder	5.4 (4.3–6.8)*	—	—
V. Any disorder	5.1 (4.3–6.0)*	—	—
Overall group effect χ^2^ (*p*-value)^d^	—	656.8 (<0.001)*	159.3 (<0.001)*

Empty cells indicate the disorder specified in the row was not included in the model.

a Bivariate models (each disorder in a separate discrete time survival model) include the following controls: age, age-squared, age cohorts, sex, and person-year.

b Multivariate additive model (all disorders together in a discrete time survival model) includes the following covariates: age, age-squared, age cohorts, sex, and person-year.

c Multivariate interactive model (all disorders together in a discrete time survival model controlling for number of disorders as interactions) includes the following covariates: age, age-squared, age cohorts, sex, and person-year.

d The group effect χ^2^ tests the set of coefficients for type of disorder for significance, while the individual χ^2^ only test presence versus absence of each DSM-IV/CIDI disorder.

*: Significant at the 0.05 level, two-sided test.

**Abbreviations:** ADHD, Attention Deficit Hyperactivity Disorder; CI, confidence interval; GAD, Generalized Anxiety Disorder; IED, Intermittent Explosive Disorder; MDD, Major Depressive Disorder; OCD, Obsessive Compulsive Disorder; ODD, Oppositional Defiant Disorder; PTSD, Posttraumatic Stress Disorder; SAD, Separation Anxiety Disorder.

### Associations of Number of Comorbid Disorders with Suicide Attempts

The model that included dummy variables for number of disorders but not types of disorders documented a strong dose-response relationship between comorbidity and risk of subsequent suicide behaviors, with ORs increasing in developed countries from 3.9 for one disorder up to 28.2 for six or more disorders (compared to respondents with no disorder) (Table 4). The same pattern was observed in developing countries, with ORs increasing from 3.5 in those with one disorder up to 32.1 in those with six or more disorders (Table 5). Notably, though, the ORs increased at a decreasing rate as number of disorders increases, which means that the *incremental* impact of each additional disorder decreased as the total number of disorders increased. This can be seen in the results of the more elaborate model that includes predictors for the 16 types of disorders (i.e., as in Tables 2 and 3) in addition to predictors for number of disorders, where the ORs associated with number of disorders were generally lower than 1.0 and became smaller as the number of disorders becomes larger. This is known as a *subadditive* interaction; that is, a situation in which the effects of comorbid disorders are less than the sum of their individual effects due to the incremental predictive power of individual disorders decaying as the number of comorbid disorders increases. It is noteworthy that even though this decay is generally monotonic, formal significance tests found that the assumption of the decay being linear can be rejected. (Detailed results are available on request.) That is why we included a series of dummy variables rather than a single continuous 0–16 measure to describe number of disorders.

**Table 4:** Survival models of the associations between number of prior DSM-IV disorders and a subsequent suicide attempt—developed countries.

	Response Variable: Lifetime Attempt among Part II Sample (*n* = 27,963)	
	Bivariate Models^a^	Multivariate Interactive Model^b^
Number of DSM-IV Disorders	OR (95% CI)	OR (95% CI)
Exactly 1	3.9 (3.2–4.7)*	—
Exactly 2	7.4 (6.0–9.1)*	1.1 (0.8–1.7)
Exactly 3	14.2 (10.9–18.3)*	1.0 (0.5–1.8)
Exactly 4	16.6 (12.6–21.8)*	0.5 (0.2–1.0)
Exactly 5	17.9 (12.7–25.4)*	0.2 (0.1–0.6)*
≥6	28.2 (20.3–39.3)*	0.1 (0.0–0.2)*
χ^2^ (*p*-value)^c^	798.4 (<0.001)*	113.5 (<0.001)*

a Bivariate discrete time survival models include the following covariates: age, age-squared, age cohort, sex, and person-year.

b Multivariate interactive model (includes number of disorders and individual DSM-IV/CIDI disorders together in a discrete time survival model) includes the following covariates: age, age-squared, age cohorts, sex, and person-year.

c The group effect χ^2^ tests the set of coefficients for number of disorders for significance, while the individual χ^2^ only test presence versus absence of each specific number of disorders.

*: Significant at the 0.05 level, two-sided test.

**Table 5:** Survival models of the associations between number of prior DSM-IV disorders and a subsequent suicide attempt—developing countries.

	Response Variable: Lifetime Attempt among Part II Sample (n = 26,959)	
	Bivariate Models^a^	Multivariate Interactive Model^b^
Number of DSM-IV Disorders	OR (95% CI)	OR (95% CI)
Exactly 1	3.5 (2.9–4.2)*	—
Exactly 2	6.8 (5.2–8.8)*	0.7 (0.5–1.0)
Exactly 3	9.4 (6.9–12.9)*	0.3 (0.2–0.6)*
Exactly 4	16.8 (11.2–25.2)*	0.2 (0.1–0.4)*
Exactly 5	19.6 (11.7–33.0)*	0.1 (0.0–0.2)*
≥6	32.1 (19.3–53.4)*	0.0 (0.0–0.0)*
χ^2^ (*p*-value)^c^	579.0 (<0.001)*	50.9 (<0.001)*

a Bivariate discrete time survival models include the following covariates: age, age-squared, age cohort, sex, and person-year.

b Multivariate interactive model (includes number of disorders and individual DSM-IV/CIDI disorders together in a discrete time survival model) includes the following covariates: age, age-squared, age cohorts, sex, and person-year.

c The group effect χ^2^ tests the set of coefficients for number of disorders for significance, while the individual χ^2^ only test presence versus absence of each specific number of disorders.

*: Significant at the 0.05 level, two-sided test. CI, confidence interval.

The model that includes the 16 dummy variables for types of disorder as well as the dummy variables for number of disorders as predictors implicitly assumes that interactions are identical for all comorbid profiles involving the same number of disorders. This assumption might be incorrect. In order to test this assumption, we also evaluated more elaborate models that considered distinct interactions for specific disorders with numbers of other disorders. However, these models did not improve over the simpler interactive model. We consequently based subsequent analyses on the simple interactive model (see Tables S3 and S4 for more detailed results).

### Unique Associations among Mental Disorders and Each Suicidal Outcome

In order to increase our understanding of how each mental disorder relates to each type of suicidal behavior, we next disaggregated the associations between each disorder and suicide attempts into five components focused on the prediction of subsequent (to the onset of the mental disorders) first onset of ideation in the total sample, onset of plans among ideators, and onset of attempts among ideators with and without a suicide plan (Tables 6 and 7). Analyses revealed that the most powerful and consistent associations are with suicide ideation (all 32 ORs are positive and statistically significant), with ORs that are relatively consistent across developed (ORs = 1.7–3.5, IQR = 1.9–2.5), and developing (ORs = 1.8–3.9, IQR = 2.3–3.4) countries. Among respondents with suicide ideation, mental disorders were much less powerful in predicting the subsequent onset of suicide plans in either developed (only eight ORs are significant, ORs = 1.0–1.6, IQR = 1.1–1.4) or developing (only seven are significant, ORs = 0.9–2.5, IQR = 1.2–1.7) countries. Mental disorders were similarly less powerful in predicting planned attempts (eight of the 16 ORs were significant in developed countries, ORs = 0.8–2.6, IQR = 1.2–1.4; only four were significant in developing countries, ORs = 1.1–3.2, IQR = 1.3–1.7) and unplanned attempts among ideators (seven of the 16 ORs were significant in developed countries, ORs = 0.7–2.2, IQR = 1.1–1.9; only two were significant in developing countries, ORs = 0.7–3.1, IQR = 0.9–1.9). In the case of comorbidity, similar to the models predicting suicide attempts in the total sample, we found ORs significantly less than 1.0 for comorbidities involving large numbers of disorders in predicting suicide ideation and planned attempts in both developed and developing countries. However, these subadditive comorbidities were not observed consistently in the prediction of plans or unplanned attempts among those with suicide ideation, which means that the multivariate effects of comorbid disorders are largely additive in predicting these outcomes.

**Table 6:** Multivariate survival models of associations between type/number of prior DSM-IV disorders and subsequent suicidal behavior—developed countries.

	Among Total Sample		Among Ideators		
	Ideation	Attempt	Plan	Planned Attempt	Unplanned Attempt
DSM-IV Disorders	OR (95% CI)	OR (95% CI)	OR (95% CI)	OR (95% CI)	OR (95% CI)
I. Anxiety disorders					
Panic disorder	1.9 (1.5–2.5)*	2.3 (1.6–3.3)*	1.3 (1.0–1.8)*	1.3 (1.0–1.9)	1.8 (1.0–3.1)*
GAD	2.5 (2.0–3.1)*	2.5 (1.9–3.4)*	1.1 (0.8–1.4)	1.4 (1.0–1.9)*	1.7 (1.1–2.6)*
Specific phobia	1.9 (1.6–2.2)*	1.9 (1.5–2.4)*	1.2 (1.0–1.4)	1.3 (1.0–1.6)*	1.3 (0.9–1.8)
Social phobia	2.3 (2.0–2.8)*	1.9 (1.4–2.5)*	1.3 (1.0–1.6)*	1.2 (0.9–1.6)	1.0 (0.6–1.4)
PTSD	2.7 (2.2–3.2)*	3.0 (2.3–3.8)*	1.4 (1.1–1.8)*	1.4 (1.1–2.0)*	2.1 (1.3–3.3)*
SAD	1.9 (1.4–2.6)*	1.6 (1.2–2.1)*	1.2 (0.9–1.6)	1.2 (0.8–1.8)	1.3 (0.8–2.2)
Agoraphobia	2.2 (1.6–2.9)*	2.2 (1.4–3.4)*	1.3 (0.9–1.9)	1.4 (0.9–2.2)	0.7 (0.3–1.8)
II. Mood disorders					
MDD	3.3 (2.8–3.9)*	3.2 (2.5–4.2)*	1.5 (1.2–1.8)*	1.5 (1.1–1.9)*	1.2 (0.8–1.7)
Dysthymia	2.1 (1.6–2.7)*	1.5 (1.0–2.2)*	1.1 (0.8–1.4)	0.8 (0.5–1.1)	1.2 (0.7–2.2)
Bipolar disorder	3.5 (2.7–4.6)*	3.5 (2.4–5.3)*	1.5 (1.1–2.0)*	2.6 (1.8–3.7)*	2.0 (1.1–3.6)*
III. Impulse–control disorders					
ODD	2.2 (1.6–2.9)*	2.2 (1.6–3.0)*	1.3 (0.9–1.9)	1.7 (1.1–2.5)*	1.9 (1.1–3.3)*
Conduct disorder	2.2 (1.6–3.0)*	2.1 (1.4–3.0)*	1.0 (0.7–1.5)	1.1 (0.7–1.7)	2.2 (1.3–3.8)*
ADHD	1.7 (1.3–2.3)*	1.9 (1.3–2.7)*	1.6 (1.1–2.2)*	1.2 (0.9–1.8)	1.6 (0.8–3.2)
IED	2.1 (1.7–2.7)*	1.8 (1.2–2.5)*	1.1 (0.8–1.6)	1.3 (1.0–1.8)*	1.0 (0.6–1.7)
IV. Substance use disorders					
Alcohol abuse or dependency	2.0 (1.7–2.4)*	2.6 (1.9–3.5)*	1.3 (1.0–1.7)*	1.3 (1.0–1.8)*	1.9 (1.2–3.1)*
Drug abuse or dependency	2.3 (1.7–3.0)*	2.0 (1.4–2.8)*	1.4 (1.1–1.9)*	1.2 (0.9–1.7)	1.0 (0.6–1.8)
V. Number of DSM-IV disorders					
Exactly 2	0.8 (0.6–1.0)	1.1 (0.8–1.7)	1.1 (0.8–1.4)	0.9 (0.7–1.3)	0.9 (0.5–1.4)
Exactly 3	0.6 (0.4–0.8)*	1.0 (0.5–1.8)	1.0 (0.7–1.5)	1.0 (0.6–1.5)	0.7 (0.3–1.6)
Exactly 4	0.3 (0.2–0.5)*	0.5 (0.2–1.0)	0.9 (0.5–1.4)	0.9 (0.5–1.5)	0.6 (0.2–1.5)
Exactly 5	0.1 (0.1–0.3)*	0.2 (0.1–0.6)*	0.6 (0.3–1.1)	0.5 (0.2–1.1)	0.4 (0.1–1.5)
≥6	0.0 (0.0–0.1)*	0.1 (0.0–0.2)*	0.6 (0.2–1.3)	0.3 (0.1–0.8)*	0.2 (0.0–1.2)
χ^2^_7_ type (*p*-value)^a^	308.6 (<.001)*	160.5 (<.001)*	31.7 (0.01)*	53.7 (<0.001)*	45.3 (<0.001)*
χ^2^_6_ number (*p*-value)^b^	150.4 (<.001)*	113.5 (<.001)*	12.5 (0.03)*	19.4 (0.002)*	6.0 (0.30)
(*n*)^c^	(27,963)	(27,963)	(4,997)	(1,874)	(3,123)

Each column includes a separate multivariate model in survival framework, with all rows as predictors controlling for the following covariates: age, age-squared, age cohort, sex, and person-year.

a χ^2^ tests for significance of the set of coefficients for type of disorder net of effects of number.

b χ^2^ tests for significance of the set of coefficients for number of disorder net of effects of type.

c Denominator sample size of the models.

*: Significant at the 0.5 level, two-sided test.

**Abbreviations:** ADHD, Attention Deficit Hyperactivity Disorder; CI, confidence interval; GAD, Generalized Anxiety Disorder; IED, Intermittent Explosive Disorder; MDD, Major Depressive Disorder; OCD, Obsessive Compulsive Disorder; ODD, Oppositional Defiant Disorder; SAD, Separation Anxiety Disorder.

**Table 7:** Multivariate survival models of associations between type/number of prior DSM-IV disorders and subsequent suicidal behavior—developing countries.

	Among Total Sample		Among Ideators		
	Ideation	Attempt	Plan	Planned Attempt	Unplanned Attempt
DSM-IV Disorders	OR (95% CI)	OR (95% CI)	OR (95% CI)	OR (95% CI)	OR (95% CI)
I. Anxiety disorders					
Panic disorder	2.5 (1.8–3.6)*	3.0 (1.7–5.4)*	1.3 (0.8–2.2)	1.9 (1.2–3.1)*	0.7 (0.2–2.4)
GAD	2.9 (2.1–4.1)*	2.9 (1.8–4.6)*	1.1 (0.8–1.7)	1.1 (0.7–1.9)	1.6 (0.6–3.9)
Specific phobia	2.2 (1.9–2.7)*	2.6 (2.0–3.4)*	1.4 (1.0–1.9)*	1.4 (1.0–1.9)	1.2 (0.8–1.8)
Social phobia	2.8 (2.2–3.5)*	2.9 (2.0–4.0)*	1.3 (0.9–1.9)	1.2 (0.7–1.9)	1.4 (0.8–2.5)
PTSD	3.9 (2.7–5.6)*	5.6 (3.5–8.8)*	2.1 (1.4–3.2)*	2.8 (1.7–4.6)*	2.0 (0.8–5.3)
OCD	1.9 (1.3–2.8)*	2.3 (1.3–4.2)*	1.0 (0.6–1.8)	1.7 (0.9–3.3)	0.8 (0.3–2.3)
SAD	2.4 (1.9–3.1)*	2.4 (1.7–3.4)*	0.9 (0.6–1.3)	1.2 (0.8–1.8)	1.6 (0.8–3.1)
Agoraphobia	1.8 (1.3–2.3)*	2.1 (1.4–3.0)*	1.3 (0.8–2.1)	1.4 (0.8–2.3)	1.7 (0.8–3.5)
II. Mood disorders					
MDD	2.9 (2.4–3.5)*	3.2 (2.4–4.3)*	1.6 (1.2–2.1)*	1.5 (1.0–2.2)*	0.7 (0.4–1.2)
Dysthymia	3.0 (2.0–4.3)*	3.5 (1.9–6.6)*	1.2 (0.7–1.9)	1.6 (1.0–2.7)	2.1 (0.7–5.8)
Bipolar disorder	3.9 (2.7–5.7)*	3.2 (1.8–5.4)*	2.5 (1.5–4.1)*	1.2 (0.6–2.4)	0.8 (0.3–2.8)
III. Impulse-control disorders					
ODD	3.2 (2.2–4.7)*	3.6 (2.0–6.5)*	2.0 (1.2–3.5)*	1.6 (0.9–2.8)	3.1 (1.3–7.3)*
Conduct disorder	3.9 (2.6–5.8)*	4.8 (2.5–9.1)*	2.0 (0.7–2.0)	3.2 (1.8–5.6)*	0.9 (0.3–2.3)
ADHD	2.2 (1.5–3.4)*	2.8 (1.6–4.9)*	1.0 (0.6–1.7)	1.7 (0.9–3.1)	1.8 (0.8–4.0)
IED	3.5 (2.7–4.3)*	3.3 (2.3–4.7)*	1.3 (1.0–1.9)	1.4 (0.9–2.1)	1.3 (0.7–2.3)
IV. Substance use disorders					
Alcohol abuse or dependency	2.5 (2.0–3.2)*	3.7 (2.6–5.4)*	1.4 (1.0–2.0)*	1.4 (1.0–2.1)	1.9 (1.1–3.5)*
Drug abuse or dependency	3.0 (2.2–4.2)*	4.0 (2.5–6.4)*	1.7 (1.1–2.6)*	1.5 (0.9–2.8)	1.4 (0.5–4.0)
V. Number of DSM-IV disorders					
Exactly 2	0.6 (0.5–0.8)*	0.7 (0.5–1.0)	1.3 (0.9–1.9)	0.8 (0.5–1.3)	0.6 (0.3–1.3)
Exactly 3	0.3 (0.2–0.5)*	0.3 (0.2–0.6)*	0.8 (0.4–1.3)	0.5 (0.3–1.0)	0.8 (0.3–2.5)
Exactly 4	0.1 (0.1–0.2)*	0.2 (0.1–0.4)*	0.7 (0.3–1.6)	0.4 (0.1–1.0)*	1.1 (0.3–4.4)
Exactly 5	0.1 (0.0–0.1)*	0.1 (0.0–0.2)*	0.9 (0.3–2.8)	0.3 (0.1–0.9)*	0.8 (0.1–5.2)
≥6	0.0 (0.0–0.0)*	0.0 (0.0–0.0)*	0.3 (0.1–1.4)	0.1 (0.0–0.6)*	0.4 (0.0–5.1)
χ^2^_17_ type (*p*-value)^a^	312.8 (<0.001)*	159.3 (<0.001)*	45.5 (<0.001)*	48.6 (<0.001)*	21.1 (0.22)
χ^2^_6_ number (*p*-value)^b^	131.1 (<0.001)*	50.9 (<0.001)*	17.5 (0.004)*	9.2 (0.10)	5.3 (0.39)
(*n*)^c^	(26,959)	(26,959))	(3,326)	(1,438)	(1,888)

Each column includes a separate multivariate model in survival framework, with all rows as predictors controlling for the following covariates: age, age-squared, age cohort, sex, and person-year.

a χ^2^ tests for significance of the set of coefficients for type of disorder net of effects of number.

b χ^2^ tests for significance of the set of coefficients for number of disorder net of effects of type.

c Denominator sample size of the models.

*: Significant at the 0.5 level, two-sided test.

**Abbreviations:** ADHD, Attention Deficit Hyperactivity Disorder; CI, confidence interval; GAD, Generalized Anxiety Disorder; IED, Intermittent Explosive Disorder; MDD, Major Depressive Disorder; OCD, Obsessive Compulsive Disorder; ODD, Oppositional Defiant Disorder; PTSD, Posttraumatic Stress Disorder; SAD, Separation Anxiety Disorder.

An examination of the predictive associations of individual disorders in developed countries revealed that depression is among the strongest predictors of suicide ideation (OR = 3.3), but is much less powerful in predicting progression from ideation to plans (OR = 1.5), planned attempts (OR = 1.5), or unplanned attempts among ideators (OR = 1.2). Instead, the strongest predictors of suicide attempts among ideators were disorders characterized by anxiety and impulse-control problems. This was especially true for unplanned attempts, for which PTSD (OR = 2.1), bipolar disorder (OR = 2.0), and conduct disorder (OR = 2.2) were the strongest predictors and the only disorders with ORs greater than 2.0.

In developing countries, depression similarly has a stronger predictive effect on ideation (OR = 2.9) than on suicide plans among ideators (OR = 1.6), planned attempts among ideators (OR = 1.5), or unplanned attempts among ideators (OR = 0.7). In these countries, a number of disorders have stronger associations with suicide ideation than depression, including: PTSD, bipolar disorder, and conduct disorder (ORs = 3.9), as well as intermittent explosive disorder (OR = 3.5), oppositional defiant disorder (OR = 3.2), drug abuse/dependence (OR = 3.0), and dysthymia (OR = 3.0). Disorders characterized by anxiety and poor impulse-control again emerged as the strongest predictors of planned attempts (PTSD, OR = 2.8; conduct disorder, OR = 3.2), and the only significant predictors of unplanned attempts (oppositional defiant disorder, OR = 3.1; alcohol abuse/dependence, OR = 1.9).

### PARPs

The findings reported so far present ORs for individual-level associations that do not take into account either the prevalence of the predictors or the distribution of comorbidity. Therefore, we calculated PARPs to test population-level effects. PARPs represent the proportion of cases with a suicide attempt that would be prevented if specified predictor variables were eliminated (i.e., prevented), assuming that the predictors are causally related to suicide attempts. For both developed and developing countries, the PARP estimates showed again that the predictive effects of mental disorders on suicide attempts are largely due to effects on ideation rather than on the transitions from ideation to plans or attempts. Focusing first on PARPs for any mental disorder, analyses showed that 59.2%–75.3% of all suicide attempts are associated with prior DSM-IV disorder. Decomposition of this effect revealed that it is due largely to the prediction of suicide ideation (60.9%–76.1%), with much smaller PARPs for the onset of a suicide plan among ideators (0.0%–4.7%), planned attempts among ideators (5.5%–18.7%), and unplanned attempts among ideators (10.2%–17.6%) (Tables 8 and 9). Mood and anxiety disorders played the largest role in accounting for the onset of ideation in both developed and developing countries. Anxiety disorders played the largest role in accounting for attempts among ideators; however, the PARPs for plans and attempts among ideators are consistently small for each class of disorder (<10%). The occasional negative PARP values associated with mood disorders in developing countries, although indicating protective effects, are so small that they are more reasonably interpreted as indicating that mood disorders have no meaningful effects on these outcomes.

**Table 8:** PARP of DSM-IV disorders—developed countries.

	Among Total Sample		Among Ideators		
DSM-IV Disorders	Ideation	Attempt	Plan	Planned Attempt	Unplanned Attempt
Any mood disorder	0.620	0.586	0.007	0.073	0.056
Any anxiety disorder	0.293	0.326	0.015	0.086	0.045
Any impulse disorder	0.072	0.064	0.003	0.009	0.029
Any substance disorder	0.132	0.135	0.015	0.022	0.064
Any disorder	0.761	0.753	0.047	0.187	0.176
(*n*)^a^	(27,963)	(27,963)	(4,997)	(1,874)	(3,123)

a Denominator sample size.

**Table 9:** PARP of DSM-IV disorders—developing countries.

	Among Total Sample		Among Ideators		
DSM-IV Disorders	Ideation	Attempt	Plan	Planned Attempt	Unplanned Attempt
Any mood disorder	0.421	0.400	−0.021	0.008	−0.031
Any anxiety disorder	0.217	0.231	0.010	0.024	0.079
Any impulse disorder	0.111	0.097	0.007	0.009	0.023
Any substance disorder	0.114	0.098	0.009	0.013	0.034
Any disorder	0.609	0.592	0.003	0.055	0.102
(*n*)^a^	(26,959)	(26,959)	(3,326)	(1,438)	(1,888)

a Denominator sample size.

## Discussion

Several noteworthy findings were revealed in this study. The finding that approximately half of those who seriously consider killing themselves, and more than half of those making a suicide attempt have a prior mental disorder extends earlier findings from psychological autopsy studies [43] and studies using clinical samples [24],[44] that have reported that most suicide attempters have a diagnosable mental disorder. Notably, the rates of mental disorder in the current study—even among suicide attempters—were much lower than those documented in prior studies among clinical samples and those dying by suicide, which suggests that the rate of mental disorders among suicidal people in the general population is lower than in these other groups. Moreover, we examined lifetime mental disorders, whereas studies of suicide decedents and clinical samples assess current or recent disorders, making the differences between clinical and general population studies even more pronounced.

Our finding of a lower rate of mental disorders among suicide attempters in the general population is consistent with other recent results indicating that prevalence estimates for suicide among those with affective disorders differ on the basis of the treatment setting from which participants are sampled (i.e., highest risk among inpatients, lower risk among outpatients, and lowest risk among the nonaffectively ill) [16]. The current study extends this finding to other diagnoses. It also is notable in the current study that the rate of mental disorders was slightly, but consistently, higher among suicidal people in developed than developing countries. However, rates of mental disorders were similarly lower among nonsuicidal people from developing countries (see Tables S1–S4), suggesting that there is a difference in the rate of mental disorders in developed versus developing countries [32], rather than in the association between disorders and suicidal behavior.

The presence of each mental disorder examined was associated with increased odds of a subsequent suicide attempt. These results extend findings from prior studies [12],[24]–[26],[45] by showing that mental disorders in general are similarly predictive of suicide attempts in developed and developing countries when similar measurement methods are used cross-nationally. We also found that while the ORs of virtually all temporally primary mental disorders predict subsequent nonfatal suicide attempts, these ORs vary significantly across disorders, demonstrating that *type* of disorder makes a difference. In addition, we found that the relative magnitudes of different types of disorders vary across stage of progression to suicide attempts (i.e., in predicting onset of ideation in the total sample, onset of plans among lifetime ideators, onset of attempts among lifetime planners, onset of attempts among lifetime ideators who never had a plan), which means that there is no single underlying common pathway (e.g., an underlying “distress” factor) through which all disorders exert their effects on the progression to attempts.

We also presented a more comprehensive analysis of the association between comorbidity and suicidal behavior than previously available, and several aspects of this association are especially noteworthy. A strong dose-response relation was found between the number of mental disorders present and the odds of a subsequent suicide attempt, consistent with the results from several prior studies [19],[24],[26],[27]. In the current study, the presence of multiple disorders (i.e., “multimorbidity” [46]) yielded ORs several times higher than that for any individual disorder in the prediction of suicide attempts. Despite this strong dose-response relation, subadditive interactive effects were observed, suggesting that decay exists in the predictive power of comorbidity as the number of comorbidities increases. This finding raises the important possibility for intervention planning purposes that the impact on reduced suicidality of successfully treating a single disorder such as major depression or panic disorder will be greater among people with pure than comorbid disorders. It also implies that success in reducing suicidality by treating people with high comorbidity will require treating multiple comorbid disorders rather than only a single component disorder.

A top priority for future research should be to understand *why* the joint predictive effects of comorbid disorders on suicidality are as powerful as they are as well as why these associations are subadditive. Prior research has shown that high comorbidity is associated with high levels of psychological distress, impairment, and disease burden [46],[47], but has shed little light on the dynamics of these associations. One possibility that has been suggested in the literature is that high comorbidity creates an especially intolerable situation from which people attempt to escape via suicide [48],[49]. However, if this were the case we would expect to find that the joint effects of comorbid conditions are supra-additive (i.e., the proverbial straw that broke the camel's back) rather than subadditive. Another possibility, as noted above in the discussion of limitations, is that we misinterpreted disorder complexity or severity as evidence of comorbidity. Future analyses examining the influence of distress, impairment, and burden on suicidal behavior should be conducted to investigate the effects of pure and comorbid disorders on these intermediate outcomes directly in order to help sort out alternative interpretations. Illumination of the psychological mechanisms through which comorbid mental disorders increase the risk of suicidal behavior is sorely needed in order to explain the causal mechanisms leading to suicide [50].

Even after accounting for the effects of comorbidity, however, each disorder considered alone continued to significantly predict subsequent suicide attempts. There has been debate regarding the extent to which some disorders appear to be associated with suicidal behavior only because they co-occur with other disorders that are truly associated with suicidal behavior. For instance, prior studies of the association between panic disorder and suicide attempts have yielded different conclusions depending on which comorbid disorders were controlled in each study [15],[22],[24]. We controlled for comorbidity more rigorously than any prior studies of which we are aware, and despite these controls panic disorder continued to significantly predict suicide attempts cross-nationally (ORs = 2.3–3.0), suggesting that panic disorder is indeed uniquely related to suicide attempts.

Consistent with results from prior studies, mood disorders—particularly major depression and bipolar disorder—were significant predictors of suicide attempts with virtually the same ORs in both developed and developing countries. Interestingly, however, several other disorders were even stronger predictors of suicide attempts in developing countries, including: conduct disorder, oppositional defiant disorder, intermittent explosive disorder, drug and alcohol abuse, and PTSD. The strong relation between impulse-control disorders and suicide attempts in developing countries extends results from a prior study using a subset of these WMH countries [19]. The strong association between PTSD and suicide attempts in developing countries is intriguing. Indeed, PTSD was the strongest predictor of suicide attempts in developing countries and was among the three strongest predictors in developed countries. A few prior studies have noted a link between PTSD and suicidal behavior [51],[52]; however, PTSD has received much less attention than mood and substance use disorders as a risk factor for suicidal behavior. This finding may be due to the lower base rate of PTSD in the population, even in developing countries and those in which war exposure is more common [53],[54], which makes this association more difficult to detect in smaller studies. Future studies should investigate whether certain types of trauma, or characteristics of traumatic events, might be particularly important in predicting suicidal behavior. Overall, these findings underscore the importance of this broader range of disorders in the onset of suicide attempts. A crucial goal for future research is to better understand how and why such a diverse range of disorders are uniquely associated with suicide attempts.

As a first step toward doing so, we found that mental disorders are differentially associated with suicidal behavior depending on which part of the pathway to suicide was being predicted. Specifically, each disorder is significantly associated with the subsequent onset of suicide ideation. However, disorders were much less useful in predicting which people with suicide ideation progress to suicide plans and attempts, and many disorders were not predictive of such progressions at all. Perhaps most surprisingly, although depression has repeatedly been shown to be among the strongest predictors of suicide attempts [13],[24],[45],[55] and was similarly predictive in the current study, decomposition of this association revealed that it is due largely to depression predicting the onset of suicide ideation. A diagnosis of major depression is much less useful in predicting which people with suicide ideation go on to make suicide plans or attempts, and it is nonsignificantly associated with unplanned attempts in both developed and developing countries. In contrast, disorders characterized by anxiety (especially PTSD) and poor impulse-control (especially bipolar, conduct, and substance use disorders) emerged as the strongest predictors of which ideators make suicide plans and attempts, and were especially useful in the prediction of unplanned attempts. These findings do not suggest that depression is unimportant in the prediction of suicidal behavior—indeed many suicide attempts occur in the context of a depressive episode—but only that a diagnosis of depression is not especially useful in determining who is likely to act on their suicidal thoughts. Several theoretical models have proposed that some disorders such as depression lead people to *desire* suicide, and other disorders characterized by anxiety/agitation and problems with impulse-control increase the likelihood that people *act* on such thoughts [56]–[60]. The current findings provide support for such a model and show that this pattern of associations is consistent cross-nationally. Future research is needed to investigate whether it is indeed the impulsiveness, aggressiveness, and agitation associated with these disorders that may lead to suicidal behavior—as has been suggested in prior studies [61]–[63]—or if some other aspects of these disorders account for the observed relations. This can be tested by examining the associations between specific symptoms of these disorders and suicidal behavior, and by testing whether the observed effects are mediated by the psychological characteristics proposed above (i.e., impulsiveness, etc.).

These results must be interpreted in light of several key limitations. First, although the WMH achieved an acceptable response rate overall, response rates varied cross-nationally. Differential response was controlled for using poststratification adjustments, but it remains possible that response rates were related to the presence of suicidal behaviors or mental disorders, which could have biased cross-national comparisons. A related limitation is that although data are from large representative samples in 21 countries, several of the samples were not nationally representative, and the WMH countries represent only a small sample from around the globe. Each of these factors limits the generality of the results, which should not be considered global estimates.

Second, these data are based on retrospective self-reports of the occurrence and timing of mental disorders and suicidal behavior, which introduce potential problems with underreporting and biased recall [64]. Recall bias is likely to be greatest for history of disorders typically diagnosed in childhood (e.g., Conduct Disorder, Oppositional Defiant Disorder, Attention Deficit Hyperactivity Disorder), which we attempted to address by limiting the diagnosis of such disorders to those <44 y old at the time of interview; however, this does not fully negate this concern. It also is possible that the extent of recall bias present differs across suicide ideation, plans, and attempts, which could have influenced the study results. On balance, systematic reviews have suggested that people can recall past experiences with sufficient accuracy to provide valuable information [65],[66] and that such data are especially useful when prospective data are not available [67], as in the current case. In addition, the WMH clinical reappraisal studies, as noted in the section on measures, found good concordance between diagnoses based on the survey responses and diagnoses based on blinded clinical follow-up interviews [38],[39]. However, this evidence is not convincing in light of the fact that the same recall bias could occur in clinical interviews. The clinical reappraisal study did not assess the concordance between survey measures of comorbidity and assessments of comorbidity in the clinical follow-up interviews. In addition, all clinical reappraisal studies were carried out in developed countries. Parallel clinical reappraisal studies need to be carried out in the future in developing countries. An additional related limitation is that cultural factors may have influenced willingness to report the presence of mental disorders or suicidal behavior [68] as well as the interpretation of survey items about these constructs [69],[70].

Third, although we assessed a broad range of disorders and various aspects of suicidal behavior, we did not consider the severity, complexity, or chronicity of each disorder. This absence could have led to an underestimate of the strength of associations between mental disorders and suicidal behavior. For instance, it may be that clinical severity and chronicity are important in predicting the transition from suicide ideation to attempts. This same problem could have led to an overestimate of comorbidity to the extent that disorder complexity or severity was incorrectly characterized as comorbidity in the CIDI. It is relevant in this regard that fully structured lay-administered diagnostic interviews like the CIDI are likely to be less capable of distinguishing between secondary symptoms of complex disorders and true comorbidity, making it likely that comorbidity is overdiagnosed and some of the presumed predictive effects of comorbidity are actually effects of disorder complexity or severity. This presumably explains why a number of respondents were found to meet criteria for six or more disorders, although the focus on lifetime hierarchy-free diagnoses also contributed to this evidence for high comorbidity. A related problem is that several disorders known to be associated with suicidal behavior were not examined in this study, such as nonaffective psychosis and personality disorders. The investigation of these and other factors remain key directions for future research.

Fourth, our analysis of comorbidity was restricted to a small number of very simple models. It is possible that more subtle interactions exist among disorders, which could be uncovered in a comprehensive analysis. Given the enormous number of logically possible interactions among the disorders considered here, it might be useful for future research to use data mining methods such as classification and regression tree analysis and random forest analysis to search for consistent patterns among these disorders [71]–[73]. Data mining has previously been used successfully to study the joint interactive effects of a wide range of risk factors for suicide attempts in a clinical sample [74]. Given the high risk of overfitting the data with methods of this sort, though, it is important that future use of these methods in the WMH data include cross-validation in random subsamples. It will also be important for such future analyses to go beyond the simple dichotomous comparison of developed versus developing countries and to evaluate the consistency of country-specific associations as well as explore plausible interpretations of meaningful cross-national differences in patterns of association.

Despite these limitations, the results of this study have important implications for scientific, clinical, and policy efforts aimed at suicide prevention. Scientifically, this study documents the importance of controlling for comorbidity and of carefully considering which suicidal behavior is being predicted in future studies. Efforts to more carefully and precisely operationalize both the independent and dependent variables in studies of suicidal behavior will yield a clearer understanding of how and why such behaviors occur. Clinically, these results demonstrate the importance of considering not only depression but also the full range of mental disorders when evaluating patients' risk for suicidal behavior. Given the especially strong associations between multimorbidity and suicidal behavior, clinicians should always conduct a suicide risk assessment among patients presenting with multiple mental disorders.

From a public health perspective, the strong and consistent associations between mental disorders and suicidal behavior suggest that suicide prevention efforts should include a focus on screening and treating mental disorders in both developed and developing countries. Resources devoted to the treatment of mental disorders in general, and suicidal behavior in particular, currently are lacking in many developing countries [30],[32],[75]. Our results suggest that if there is in fact a causal relation between mental disorders and suicide attempts, allocating sufficient resources to decrease mental disorders will lead to a significant reduction in suicidal behavior. Although it is not yet known whether a causal association exists, there is some evidence that efforts to decrease the occurrence of mental disorders can positively affect the suicide rate. For instance, some programs designed to train primary care physicians in the recognition and treatment of depression have demonstrated reductions in the suicide rate [76]–[78]; however, not all such programs have yielded positive effects [79]. Importantly, though, our results also indicate that a sizeable minority of respondents report suicidal behaviors in the absence of *any* mental disorder. Thus, focusing solely on those with mental disorders is likely to miss a fairly large segment of those who engage in suicidal behavior. Identifying this subgroup is likely to be more challenging given that public health programs that screen for the presence of mental disorders will not identify these respondents, highlighting the need for novel methods of identifying those at-risk that do not rely on the presence of mental disorders [80]. There is still much we do not know about how mental disorders and other factors increase the risk of suicidal behavior; however, the results of this and related studies highlight several important directions for improving the understanding, prediction, and prevention of these dangerous outcomes.

## Supporting Information

Table S1 Prevalence of lifetime DSM-IV disorders among suicidality in developed countries.
(0.02 MB PDF)

Table S2 Prevalence of lifetime DSM-IV disorders among suicidality in developing countries.
(0.02 MB PDF)

Table S3 Multivariate survival models of interactive associations between type and number of temporally primary lifetime DSM-IV/CIDI disorders in predicting the subsequent first occurrence of suicidal behaviors—developed countries.
(0.02 MB PDF)

Table S4 Multivariate survival models of interactive associations between type and number of temporally primary lifetime DSM-IV/CIDI disorders in predicting the subsequent first occurrence of suicidal behaviors—developing countries.
(0.02 MB PDF)

## Abbreviations

CIDI: Composite International Diagnostic Interview
DSM-IV: Diagnostic and Statistical Manual of Mental Disorders, 4th Edition
IQR: interquartile range
OR: odds ratio
PARP: population attributable risk proportion
PTSD: posttraumatic stress
WMH: WHO World Mental Health
