# Supplementary material for: Cross-National Analysis of the Associations among Mental Disorders and Suicidal Behavior: Findings from the WHO World Mental Health Surveys
Source: PLoS Med. 2009 Aug 11;6(8):e1000123. doi: 10.1371/journal.pmed.1000123 (PMC2717212; doi:10.1371/journal.pmed.1000123)
Supplement: Table S1 — Prevalence of lifetime DSM-IV disorders among suicidality in developed countries. (0.02 MB PDF) [file pmed.1000123.s001.pdf]

**Table S2. Prevalence of lifetime DSM-IV disorders among suicidality in developing countries**

| | Among total sample | | | | | | Among total sample | | | | | | Among Ideators | | | | | | Among ideators with a lifetime plan | | | | | | Among ideators without a lifetime plan | | | | | |
|---|---|---|---|---|---|---|---|---|---|---|---|---|---|---|---|---|---|---|---|---|---|---|---|---|---|---|---|---|---|---|
| | % with disorder among | | | | | | % with disorder among | | | | | | % with disorder among | | | | | | % with disorder among | | | | | | % with disorder among | | | | | |
| | Ideation | | | No ideation | | | Attempt | | | No attempt | | | Plan | | | No plan | | | Attempt | | | No attempt | | | Attempt | | | No attempt | | |
| | N[2] | %[3] | SE | N[2] | %[3] | SE | N[2] | %[3] | SE | N[2] | %[3] | SE | N[2] | %[3] | SE | N[2] | %[3] | SE | N[2] | %[3] | SE | N[2] | %[3] | SE | N[2] | %[3] | SE | N[2] | %[3] | SE |
| I. Anxiety Disorders | | | | | | | | | | | | | | | | | | | | | | | | | | | | | | |
| Panic Disorder[4] | 85 | 2.0 | 0.3 | 442 | 0.7 | 0.1 | 40 | 3.1 | 0.6 | 532 | 0.8 | 0.1 | 48 | 3.1 | 0.5 | 81 | 2.8 | 0.4 | 35 | 4.0 | 0.8 | 20 | 3.1 | 0.8 | 5 | 1.3 | 0.6 | 70 | 2.8 | 0.4 |
| General Anxiety Disorder[4] | 129 | 3.2 | 0.4 | 826 | 1.4 | 0.1 | 63 | 5.0 | 0.7 | 1013 | 1.7 | 0.1 | 64 | 4.8 | 0.7 | 159 | 5.7 | 0.6 | 41 | 5.5 | 1.0 | 64 | 11.7 | 1.7 | 22 | 4.0 | 1.0 | 123 | 5.3 | 0.7 |
| Specific Phobia[4] | 571 | 13.7 | 0.7 | 2689 | 5.1 | 0.1 | 248 | 19.3 | 1.4 | 3051 | 5.5 | 0.1 | 282 | 18.8 | 1.5 | 326 | 12.4 | 0.8 | 188 | 21.4 | 1.9 | 102 | 17.4 | 1.9 | 60 | 15.0 | 2.2 | 260 | 11.7 | 0.8 |
| Social Phobia[4] | 250 | 6.2 | 0.5 | 839 | 1.6 | 0.1 | 105 | 8.7 | 1.0 | 1021 | 1.9 | 0.1 | 119 | 8.7 | 1.0 | 158 | 6.0 | 0.5 | 76 | 9.3 | 1.3 | 56 | 10.4 | 1.7 | 29 | 7.7 | 1.6 | 126 | 5.6 | 0.6 |
| Obsessive-Compulsive Disorder[9] | 102 | 3.3 | 0.5 | 513 | 1.3 | 0.1 | 62 | 5.8 | 0.8 | 624 | 1.5 | 0.1 | 66 | 5.0 | 0.7 | 93 | 5.1 | 0.7 | 48 | 6.8 | 1.1 | 39 | 6.8 | 1.4 | 14 | 3.7 | 1.3 | 72 | 5.0 | 0.8 |
| Post-Traumatic Disorder[5] | 81 | 2.8 | 0.4 | 447 | 1.5 | 0.1 | 27 | 3.3 | 0.8 | 548 | 1.7 | 0.1 | 36 | 3.4 | 0.8 | 90 | 4.9 | 0.6 | 22 | 3.6 | 1.0 | 24 | 5.2 | 1.6 | 5 | 2.6 | 1.5 | 77 | 4.9 | 0.7 |
| Separation Anxiety Disorder[6] | 205 | 6.3 | 0.5 | 774 | 2.6 | 0.1 | 93 | 8.4 | 1.1 | 940 | 2.9 | 0.1 | 100 | 7.2 | 0.8 | 160 | 8.8 | 0.9 | 58 | 7.7 | 1.3 | 52 | 8.5 | 1.4 | 35 | 9.8 | 2.2 | 114 | 7.8 | 1.0 |
| Agoraphobia[4] | 119 | 2.6 | 0.3 | 636 | 1.2 | 0.1 | 55 | 3.7 | 0.6 | 733 | 1.3 | 0.1 | 58 | 3.7 | 0.6 | 92 | 3.1 | 0.4 | 38 | 3.9 | 0.7 | 25 | 4.7 | 1.2 | 17 | 3.4 | 1.0 | 72 | 2.9 | 0.4 |
| Any Anxiety Disorder[5] | 1020 | 27.1 | 0.9 | 4661 | 12.0 | 0.2 | 439 | 36.3 | 1.8 | 5450 | 13.0 | 0.2 | 512 | 35.3 | 1.7 | 696 | 30.4 | 1.3 | 323 | 39.9 | 2.6 | 242 | 40.3 | 2.7 | 116 | 29.3 | 2.9 | 547 | 29.2 | 1.4 |
| II. Mood Disorders | | | | | | | | | | | | | | | | | | | | | | | | | | | | | | |
| Major Depression[4] | 560 | 12.9 | 0.6 | 3846 | 6.8 | 0.2 | 243 | 18.2 | 1.3 | 4679 | 7.9 | 0.2 | 306 | 20.4 | 1.3 | 666 | 24.9 | 1.0 | 187 | 21.6 | 1.7 | 272 | 43.0 | 2.6 | 56 | 11.5 | 1.7 | 561 | 24.6 | 1.1 |
| Dysthymia[4] | 118 | 2.8 | 0.3 | 567 | 0.9 | 0.1 | 60 | 4.6 | 0.8 | 690 | 1.1 | 0.1 | 63 | 4.0 | 0.6 | 106 | 4.1 | 0.5 | 45 | 5.3 | 1.0 | 40 | 6.4 | 1.1 | 15 | 3.3 | 1.0 | 83 | 3.9 | 0.5 |
| Bipolar[4] | 64 | 1.7 | 0.3 | 350 | 0.8 | 0.1 | 30 | 2.4 | 0.5 | 450 | 0.9 | 0.1 | 44 | 3.3 | 0.7 | 74 | 2.9 | 0.4 | 27 | 3.3 | 0.7 | 38 | 7.2 | 1.4 | 3 | 0.7 | 0.4 | 62 | 2.8 | 0.5 |
| Any Mood[4] | 607 | 13.9 | 0.6 | 4162 | 7.5 | 0.2 | 260 | 19.4 | 1.3 | 5052 | 8.6 | 0.2 | 333 | 22.0 | 1.3 | 706 | 26.4 | 0.9 | 203 | 23.3 | 1.8 | 293 | 46.5 | 2.6 | 57 | 11.6 | 1.7 | 597 | 26.1 | 1.0 |
| III. Impulse-Control Disorders | | | | | | | | | | | | | | | | | | | | | | | | | | | | | | |
| ODD[7] | 79 | 3.0 | 0.5 | 146 | 0.5 | 0.1 | 47 | 5.0 | 1.0 | 179 | 0.6 | 0.1 | 46 | 4.6 | 0.9 | 36 | 2.2 | 0.4 | 35 | 5.3 | 1.3 | 10 | 3.5 | 1.3 | 12 | 4.2 | 1.4 | 23 | 1.8 | 0.5 |
| Conduct[7] | 86 | 3.2 | 0.5 | 137 | 0.5 | 0.1 | 53 | 5.9 | 1.1 | 174 | 0.6 | 0.1 | 48 | 4.8 | 0.9 | 42 | 2.6 | 0.6 | 38 | 6.8 | 1.4 | 10 | 2.3 | 0.9 | 15 | 4.2 | 1.3 | 27 | 2.3 | 0.6 |
| ADD[7] | 80 | 2.5 | 0.4 | 148 | 0.5 | 0.1 | 40 | 3.7 | 0.8 | 188 | 0.6 | 0.1 | 44 | 3.0 | 0.5 | 36 | 2.1 | 0.5 | 29 | 3.5 | 0.8 | 15 | 2.4 | 0.7 | 11 | 4.2 | 1.5 | 25 | 1.8 | 0.5 |
| IED[4] | 250 | 6.5 | 0.5 | 917 | 2.0 | 0.1 | 105 | 8.8 | 1.1 | 1148 | 2.3 | 0.1 | 128 | 9.8 | 1.0 | 210 | 8.2 | 0.7 | 74 | 9.7 | 1.3 | 72 | 12.6 | 1.7 | 31 | 7.1 | 1.5 | 159 | 7.5 | 0.7 |
| Any Impulse[7] | 358 | 13.2 | 1.0 | 968 | 3.8 | 0.2 | 172 | 19.0 | 1.8 | 1218 | 4.3 | 0.2 | 196 | 19.7 | 1.5 | 235 | 13.7 | 1.1 | 125 | 20.8 | 2.3 | 78 | 18.8 | 2.6 | 47 | 15.3 | 2.4 | 172 | 12.7 | 1.2 |
| IV. Substance Use Disorders | | | | | | | | | | | | | | | | | | | | | | | | | | | | | | |
| Alcohol Abuse or Dependence[4] | 267 | 7.9 | 0.6 | 2438 | 5.6 | 0.2 | 106 | 10.5 | 1.1 | 2757 | 6.0 | 0.2 | 137 | 11.1 | 1.2 | 282 | 13.1 | 0.9 | 79 | 10.6 | 1.4 | 103 | 20.4 | 2.2 | 27 | 10.3 | 2.3 | 216 | 11.8 | 1.0 |
| Drug Abuse or Dependence[8] | 90 | 3.1 | 0.4 | 332 | 1.1 | 0.1 | 52 | 4.9 | 0.7 | 421 | 1.4 | 0.1 | 52 | 4.6 | 0.7 | 76 | 5.3 | 0.7 | 44 | 5.8 | 0.9 | 31 | 8.8 | 2.5 | 8 | 3.0 | 1.1 | 58 | 5.0 | 0.8 |
| Any Substance Disorder[8] | 292 | 9.7 | 0.7 | 2107 | 6.7 | 0.2 | 128 | 13.3 | 1.3 | 2433 | 7.3 | 0.2 | 161 | 13.6 | 1.4 | 281 | 15.9 | 1.2 | 99 | 14.0 | 1.6 | 116 | 25.0 | 3.1 | 29 | 11.8 | 2.6 | 210 | 14.5 | 1.3 |
| Any Disorder[10] | 1611 | 42.9 | 1.1 | 8703 | 21.8 | 0.3 | 666 | 54.6 | 2.0 | 10112 | 23.6 | 0.3 | 804 | 55.5 | 1.7 | 1219 | 53.0 | 1.5 | 489 | 59.6 | 2.4 | 434 | 69.6 | 2.7 | 177 | 44.6 | 3.2 | 975 | 51.5 | 1.7 |
| V. Number of Disorders | | | | | | | | | | | | | | | | | | | | | | | | | | | | | | |
| Exactly 1[10] | 852 | 23.3 | 0.9 | 5408 | 14.4 | 0.3 | 311 | 25.7 | 1.6 | 6034 | 15.0 | 0.3 | 385 | 25.6 | 1.4 | 546 | 24.9 | 1.4 | 225 | 27.0 | 1.9 | 178 | 25.1 | 2.5 | 86 | 23.3 | 2.9 | 448 | 24.9 | 1.5 |
| Exactly 2[10] | 402 | 10.6 | 0.8 | 1980 | 4.6 | 0.2 | 163 | 13.5 | 1.5 | 2380 | 5.1 | 0.2 | 213 | 15.8 | 1.4 | 339 | 14.0 | 0.9 | 123 | 15.9 | 2.0 | 120 | 20.2 | 1.9 | 40 | 8.7 | 1.6 | 280 | 13.7 | 1.0 |
| Exactly 3[10] | 180 | 4.7 | 0.5 | 795 | 1.8 | 0.1 | 82 | 6.5 | 0.9 | 985 | 2.1 | 0.1 | 92 | 5.9 | 0.7 | 175 | 7.5 | 0.8 | 55 | 7.0 | 1.2 | 63 | 10.1 | 1.6 | 27 | 5.6 | 1.3 | 127 | 7.1 | 0.9 |
| Exactly 4[10] | 88 | 2.2 | 0.3 | 311 | 0.6 | 0.1 | 52 | 4.3 | 0.7 | 403 | 0.8 | 0.1 | 55 | 4.2 | 0.7 | 75 | 3.7 | 0.6 | 41 | 4.7 | 0.9 | 35 | 6.1 | 1.4 | 11 | 3.6 | 1.3 | 57 | 3.3 | 0.5 |
| Exactly 5[10] | 44 | 1.1 | 0.2 | 127 | 0.2 | 0.0 | 28 | 1.9 | 0.4 | 175 | 0.3 | 0.0 | 30 | 2.3 | 0.5 | 39 | 1.3 | 0.3 | 22 | 2.4 | 0.6 | 20 | 4.3 | 1.5 | 6 | 1.0 | 0.5 | 28 | 1.1 | 0.3 |
| 6 or more disorders[10] | 45 | 1.0 | 0.2 | 82 | 0.2 | 0.0 | 30 | 2.6 | 0.6 | 135 | 0.3 | 0.0 | 29 | 1.8 | 0.4 | 45 | 1.7 | 0.3 | 23 | 2.7 | 0.7 | 18 | 3.9 | 1.4 | 7 | 2.4 | 1.0 | 35 | 1.4 | 0.3 |
| (N)[1] | (4090) | | | (52803) | | | (1223) | | | (55670) | | | (1443) | | | (2647) | | | (835) | | | (608) | | | (388) | | | (2259) | | |

[1] Number of cases with the outcome variable

[2] Numerator N. Number of cases with the LT disorder among cases with the outcome variable. Columns with "Yes" in the headers represents cases with the dx among those with the outcome, and columns with "No" represents among those without the outcome. Part II disorders assessed in part II sample, and disorders with age restrictions restricted to appropriate age range

[3] % represents the percentage of people with the DSM-IV disorder among the cases with the outcome variable indicated in the column header. For example: the first cell is the % of those with Panic disorders among those with attempts

[4] assessed in part I sample

[5] assessed in part II sample

[6] assessed in part II sample. Restricted to age groups 18-44 for Lebanon and India, 18-39 for China and Nigeria, and not restricted for other countries.

[7] assessed in part II sample and restricted to age groups 18-44 for Mexico, Colombia, Bulgaria, India, 18-39 for China, Nigeria, Ukraine, and not restricted for Brazil and Romania.

[8] assessed in part II sample for all data except Colombia and Mexico, where part I weight is used. A weight variable is created that takes the part I weight values for Colombia and Mexico and part II for other countries.

[9] OCD was only assessed in Brazil, Bulgaria, India, Romania, and Shenzhen as there was a problem with the variable in other countries. Assessed in part I sample for Shenzhen and India while in part II sample for Brazil, Bulgaria, and Romania.

[10] assessed in part II sample for all data except for Shenzhen, where part I weight is used.
