# Supplementary material for: Cross-National Analysis of the Associations among Mental Disorders and Suicidal Behavior: Findings from the WHO World Mental Health Surveys
Source: PLoS Med. 2009 Aug 11;6(8):e1000123. doi: 10.1371/journal.pmed.1000123 (PMC2717212; doi:10.1371/journal.pmed.1000123)
Supplement: Table S2 — Prevalence of lifetime DSM-IV disorders among suicidality in developing countries. (0.02 MB PDF) [file pmed.1000123.s002.pdf]

Table S3. Multivariate survival models of interactive associations between type and number of temporally primary lifetime DSM-IV/CIDI disorders in predicting the subsequent first occurrence of suicidal behaviors—Developed Countries[a]

	Ideation		Attempt		Plan among Ideators		Planned Attempt: Attempt among ideators with a lifetime plan		Unplanned Attempt: Attempt among ideators without a lifetime plan	
	OR (95% CI)	Chisquare	OR (95% CI)	Chisquare	OR (95% CI)	Chisquare	OR (95% CI)	Chisquare	OR (95% CI)	Chisquare
I. Anxiety Disorders										
Panic Disorder	2.43 (1.63-3.62)*	19.2(<.001)*	4.03 (2.09-7.79)*	17.3(<.001)*	1.67 (1.06-2.63)*	4.8(0.028)*	0.95 (0.52-1.74)	0.0(0.88)	4.26 (1.62-11.17)*	8.7(0.003)*
GAD	4.11 (3.16-5.35)*	110.6(<.001)*	5.02 (3.38-7.46)*	64.0(<.001)*	1.30 (0.89-1.89)	1.8(0.18)	1.60 (0.98-2.61)	3.5(0.06)	1.72 (0.86-3.44)	2.4(0.13)
Specific Phobia	1.73 (1.50-1.99)*	56.8(<.001)*	2.32 (1.81-2.98)*	43.5(<.001)*	1.30 (1.04-1.61)*	5.6(0.018)*	1.20 (0.88-1.63)	1.3(0.25)	1.45 (1.01-2.09)*	4.0(0.046)*
Social Phobia	2.47 (2.11-2.89)*	124.9(<.001)*	2.57 (1.92-3.43)*	40.9(<.001)*	1.32 (1.01-1.72)*	4.2(0.041)*	1.66 (1.15-2.41)*	7.2(0.007)*	0.65 (0.40-1.06)	3.0(0.08)
PTSD	3.09 (2.35-4.07)*	64.8(<.001)*	4.47 (3.06-6.54)*	59.6(<.001)*	1.67 (1.18-2.38)*	8.3(0.004)*	1.97 (1.22-3.18)*	7.8(0.005)*	2.17 (1.17-4.01)*	6.0(0.014)*
SAD	2.40 (1.61-3.60)*	18.1(<.001)*	2.48 (1.51-4.07)*	12.9(<.001)*	0.91 (0.53-1.56)	0.1(0.74)	1.28 (0.64-2.54)	0.5(0.49)	1.26 (0.51-3.16)	0.3(0.62)
Agoraphobia	2.29 (1.35-3.87)*	9.5(0.002)*	3.07 (1.16-8.14)*	5.1(0.024)*	2.16 (1.10-4.24)*	5.0(0.025)*	1.31 (0.58-2.93)	0.4(0.51)	0.79 (0.11-5.42)	0.1(0.81)
II. Mood Disorders										
MDD	4.02 (3.39-4.75)*	261.8(<.001)*	4.56 (3.43-6.07)*	108.8(<.001)*	1.41 (1.09-1.81)*	6.9(0.009)*	1.59 (1.16-2.16)*	8.5(0.004)*	1.33 (0.87-2.02)	1.8(0.18)
Dysthymia	2.92 (1.98-4.30)*	29.4(<.001)*	2.34 (1.20-4.56)*	6.2(0.013)*	1.15 (0.65-2.04)	0.2(0.63)	0.65 (0.31-1.34)	1.4(0.24)	1.11 (0.29-4.21)	0.0(0.88)
Bipolar Disorder	5.93 (4.17-8.43)*	98.4(<.001)*	8.19 (4.66-14.39)*	53.5(<.001)*	2.68 (1.69-4.26)*	17.6(<.001)*	3.36 (1.99-5.66)*	20.7(<.001)*	2.92 (1.23-6.97)*	5.9(0.015)*
III. Impulse-Control Disorders										
ODD	3.58 (2.43-5.29)*	41.2(<.001)*	2.98 (1.53-5.81)*	10.2(0.001)*	0.84 (0.39-1.78)	0.2(0.64)	1.23 (0.45-3.34)	0.2(0.69)	1.80 (0.64-5.00)	1.3(0.26)
Conduct Disorder	3.26 (2.30-4.62)*	43.8(<.001)*	2.94 (1.64-5.29)*	13.0(<.001)*	1.71 (0.82-3.57)	2.0(0.16)	0.95 (0.33-2.70)	0.0(0.92)	1.37 (0.48-3.94)	0.3(0.56)
ADD	2.21 (1.41-3.46)*	12.0(<.001)*	4.01 (1.98-8.11)*	14.9(<.001)*	2.93 (1.31-6.54)*	6.9(0.009)*	1.59 (0.68-3.69)	1.1(0.28)	1.66 (0.64-4.28)	1.1(0.29)
IED	2.63 (1.74-3.96)*	21.3(<.001)*	2.49 (1.50-4.13)*	12.4(<.001)*	0.83 (0.51-1.34)	0.6(0.44)	1.42 (0.67-3.01)	0.9(0.35)	1.21 (0.53-2.79)	0.2(0.65)
IV. Substance Abuse Disorders										
Alcohol abuse or dependence	1.89 (1.49-2.40)*	27.0(<.001)*	3.18 (2.16-4.68)*	34.6(<.001)*	1.46 (1.01-2.10)*	4.1(0.042)*	1.41 (0.85-2.36)	1.7(0.19)	1.86 (1.01-3.45)*	3.9(0.048)*
drug abuse or dependence	3.19 (2.14-4.76)*	32.2(<.001)*	4.11 (2.45-6.90)*	28.6(<.001)*	1.79 (1.14-2.80)*	6.5(0.011)*	1.36 (0.67-2.75)	0.7(0.40)	2.36 (1.06-5.26)*	4.4(0.035)*
continuous variable for # of other disorders[b]	0.83 (0.72-0.96)*	6.3(0.012)*	0.86 (0.71-1.05)	2.2(0.14)	0.94 (0.77-1.14)	0.4(0.53)	1.03 (0.82-1.30)	0.1(0.79)	0.81 (0.57-1.16)	1.3(0.25)
**Interactions with Number of Other Disorders**										
I. Anxiety Disorders										
Panic Disorder	0.88 (0.77-1.02)	2.9(0.09)	0.85 (0.70-1.03)	2.9(0.09)	0.93 (0.81-1.08)	0.9(0.35)	1.12 (0.94-1.34)	1.7(0.19)	0.67 (0.46-0.99)*	4.0(0.046)*
GAD	0.77 (0.70-0.85)*	26.7(<.001)*	0.78 (0.68-0.90)*	12.5(<.001)*	0.93 (0.82-1.05)	1.4(0.23)	0.93 (0.79-1.08)	1.0(0.33)	1.01 (0.78-1.30)	0.0(0.95)
Specific Phobia	1.06 (0.98-1.15)	2.4(0.12)	0.95 (0.84-1.07)	0.6(0.42)	0.98 (0.89-1.07)	0.2(0.63)	1.03 (0.90-1.19)	0.2(0.64)	0.93 (0.80-1.09)	0.8(0.37)
Social Phobia	0.95 (0.86-1.04)	1.2(0.26)	0.91 (0.81-1.02)	2.6(0.11)	1.01 (0.91-1.13)	0.1(0.82)	0.82 (0.72-0.94)*	8.2(0.004)*	1.29 (1.06-1.58)*	6.4(0.012)*
PTSD	0.90 (0.82-1.00)	3.8(0.05)	0.87 (0.76-0.99)*	4.5(0.034)*	0.95 (0.84-1.06)	0.9(0.34)	0.85 (0.73-0.99)*	4.4(0.037)*	0.99 (0.81-1.21)	0.0(0.91)
SAD	0.88 (0.78-0.98)*	5.3(0.021)*	0.87 (0.75-1.02)	2.9(0.09)	1.09 (0.88-1.35)	0.6(0.43)	0.97 (0.81-1.17)	0.1(0.78)	1.00 (0.74-1.35)	0.0(0.99)
Agoraphobia	0.91 (0.77-1.08)	1.1(0.29)	0.88 (0.66-1.18)	0.7(0.40)	0.82 (0.66-1.02)	3.3(0.07)	1.04 (0.81-1.35)	0.1(0.75)	0.90 (0.54-1.51)	0.2(0.70)
II. Mood Disorders										
MDD	0.84 (0.77-0.90)*	21.8(<.001)*	0.85 (0.77-0.94)*	9.5(0.002)*	1.05 (0.93-1.18)	0.7(0.41)	0.95 (0.82-1.10)	0.5(0.48)	0.93 (0.78-1.11)	0.7(0.40)
Dysthymia	0.89 (0.77-1.02)	2.7(0.10)	0.91 (0.75-1.10)	1.0(0.31)	0.96 (0.82-1.13)	0.2(0.66)	1.07 (0.86-1.33)	0.3(0.56)	1.04 (0.73-1.49)	0.1(0.81)
Bipolar Disorder	0.78 (0.69-0.88)*	15.6(<.001)*	0.75 (0.63-0.88)*	12.7(<.001)*	0.80 (0.70-0.93)*	8.8(0.003)*	0.90 (0.75-1.07)	1.6(0.21)	0.84 (0.63-1.11)	1.5(0.22)
III. Impulse-Control Disorders										
ODD	0.79 (0.68-0.91)*	11.0(<.001)*	0.92 (0.73-1.15)	0.6(0.45)	1.23 (0.97-1.57)	2.8(0.09)	1.08 (0.78-1.48)	0.2(0.65)	1.03 (0.73-1.44)	0.0(0.89)
Conduct Disorder	0.84 (0.75-0.96)*	7.2(0.007)*	0.92 (0.74-1.13)	0.6(0.43)	0.83 (0.67-1.02)	3.1(0.08)	1.04 (0.79-1.38)	0.1(0.78)	1.24 (0.89-1.72)	1.6(0.21)
ADD	0.91 (0.79-1.05)	1.6(0.21)	0.78 (0.61-0.99)*	4.1(0.042)*	0.77 (0.57-1.04)	2.9(0.09)	0.90 (0.67-1.20)	0.5(0.47)	0.96 (0.76-1.20)	0.1(0.71)
IED	0.89 (0.76-1.04)	2.2(0.14)	0.90 (0.76-1.07)	1.4(0.24)	1.13 (0.97-1.32)	2.5(0.12)	0.95 (0.75-1.21)	0.2(0.70)	0.91 (0.72-1.14)	0.7(0.39)
IV. Substance Abuse Disorders										
Alcohol abuse or dependence	1.03 (0.92-1.14)	0.2(0.63)	0.94 (0.82-1.08)	0.8(0.37)	0.96 (0.84-1.09)	0.4(0.52)	0.97 (0.80-1.17)	0.1(0.74)	1.07 (0.84-1.35)	0.3(0.60)
drug abuse or dependence	0.83 (0.72-0.95)*	7.2(0.007)*	0.78 (0.66-0.93)*	7.6(0.006)*	0.93 (0.81-1.06)	1.1(0.29)	0.95 (0.77-1.16)	0.3(0.58)	0.66 (0.50-0.88)*	8.0(0.005)*
df interaction test[c]		228.2(<.001)*		175.4(<.001)*		40.6(<.001)*		32.2(0.010)*		23.5(0.10)
(N)[d]	(27963)		(27963)		(4997)		(1874)		(3123)	

Abbreviations: GAD, Generalized Anxiety Disorder; PTSD, Posttraumatic Stress Disorder; OCD, Obsessive Compulsive Disorder; SAD, Separation Anxiety Disorder; MDD, Major Depressive Disorder; ODD, Oppositional Defiant Disorder; ADHD, Attention Deficit Hyperactivity Disorder; IED, Intermittent Explosive Disorder.

* Significant at the .05 level, two-sided test

[a]Each column includes a separate multivariate model in survival framework, with all rows as predictors controlling for the following covariates: age, age-squared, age cohort, sex, and person-year.

[b]Number of other disorders represents the number of disorders in addition to the first one counted, so cases with 1 disorder will have 0 other disorders, cases with 2 disorders will have 1 other disorders, and so on. This is used in the multivariate model because the individual disorders are also in the model, and including a continuous number of disorder from 0,1,2,... will cause the model to overfit.

[c]Multi-df tests assess the overall interactions. This group effect test only includes all of the interactions and not all of the independent variables.

[d]Denominator sample size of the models.
