# Supplementary material for: Cross-National Analysis of the Associations among Mental Disorders and Suicidal Behavior: Findings from the WHO World Mental Health Surveys
Source: PLoS Med. 2009 Aug 11;6(8):e1000123. doi: 10.1371/journal.pmed.1000123 (PMC2717212; doi:10.1371/journal.pmed.1000123)
Supplement: Table S3 — Multivariate survival models of interactive associations between type and number of temporally primary lifetime DSM-IV/CIDI disorders in predicting the subsequent first occurrence of suicidal behaviors—developed countries. (0.02 MB PDF) [file pmed.1000123.s003.pdf]

Table S4. Multivariate survival models of interactive associations between type-number of temporally primary lifetime DSM-IV/CIDI disorders in predicting the subsequent first occurrence of suicidal behaviors—Developing Countries [a]

	Ideation		Attempt		Plan among Ideators		Planned Attempt: Attempt among ideators with a lifetime plan		Unplanned Attempt: Attempt among ideators without a lifetime plan	
	OR (95% CI)	Chisquare	OR (95% CI)	Chisquare	OR (95% CI)	Chisquare	OR (95% CI)	Chisquare	OR (95% CI)	Chisquare
I. Anxiety Disorders										
Panic Disorder	2.50 (1.58-3.94)*	15.5(<.001)*	4.43 (2.17-9.04)*	16.7(<.001)*	1.34 (0.62-2.90)	0.6(0.45)	2.14 (1.14-4.03)*	5.6(0.018)*	1.57 (0.45-5.55)	0.5(0.48)
GAD	2.07 (1.22-3.52)*	7.3(0.007)*	3.54 (1.73-7.23)*	12.1(<.001)*	1.04 (0.47-2.34)	0.0(0.92)	1.39 (0.62-3.09)	0.6(0.42)	1.84 (0.51-6.68)	0.9(0.35)
Specific Phobia	2.17 (1.81-2.59)*	71.6(<.001)*	2.55 (1.93-3.37)*	43.3(<.001)*	1.56 (1.15-2.11)*	8.1(0.004)*	1.34 (0.92-1.96)	2.3(0.13)	1.16 (0.73-1.84)	0.4(0.53)
Social Phobia	2.86 (1.99-4.09)*	33.0(<.001)*	2.64 (1.49-4.67)*	11.2(<.001)*	0.92 (0.54-1.58)	0.1(0.77)	1.37 (0.72-2.63)	0.9(0.34)	1.04 (0.45-2.40)	0.0(0.93)
PTSD	4.86 (3.05-7.73)*	44.7(<.001)*	8.37 (4.46-15.71)*	44.0(<.001)*	2.81 (1.50-5.27)*	10.5(0.001)*	5.46 (2.95-10.14)*	29.1(<.001)*	2.13 (0.46-9.86)	0.9(0.33)
OCD	1.63 (0.98-2.72)	3.5(0.06)	1.66 (0.69-4.00)	1.3(0.26)	0.88 (0.36-2.19)	0.1(0.79)	1.19 (0.35-3.97)	0.1(0.78)	0.67 (0.07-6.18)	0.1(0.73)
SAD	2.27 (1.66-3.10)*	26.9(<.001)*	2.07 (1.26-3.42)*	8.2(0.004)*	0.88 (0.55-1.42)	0.3(0.61)	0.95 (0.56-1.60)	0.0(0.83)	1.16 (0.48-2.81)	0.1(0.74)
Agoraphobia	1.25 (0.84-1.86)	1.3(0.26)	1.49 (0.86-2.58)	2.0(0.16)	1.34 (0.84-2.14)	1.5(0.21)	2.02 (1.04-3.91)*	4.3(0.038)*	1.74 (0.56-5.38)	0.9(0.33)
II. Mood Disorders										
MDD	3.13 (2.52-3.89)*	107.1(<.001)*	3.46 (2.44-4.90)*	48.8(<.001)*	1.94 (1.44-2.62)*	18.9(<.001)*	1.38 (0.91-2.09)	2.4(0.12)	0.68 (0.35-1.30)	1.4(0.24)
Dysthymia	3.87 (2.24-6.68)*	23.7(<.001)*	4.36 (1.95-9.74)*	12.9(<.001)*	2.20 (1.13-4.28)*	5.4(0.020)*	1.39 (0.56-3.43)	0.5(0.48)	1.05 (0.18-6.01)	0.0(0.96)
Bipolar Disorder	4.44 (2.78-7.07)*	39.4(<.001)*	3.96 (1.66-9.47)*	9.7(0.002)*	7.66 (3.70-15.87)*	30.2(<.001)*	0.75 (0.18-3.15)	0.2(0.70)	0.56 (0.07-4.69)	0.3(0.60)
III. Impulse-Control Disorders										
ODD	4.04 (2.49-6.58)*	31.9(<.001)*	5.70 (2.78-11.70)*	22.6(<.001)*	2.65 (1.19-5.90)*	5.7(0.017)*	1.51 (0.59-3.86)	0.7(0.39)	2.92 (0.69-12.41)	2.1(0.14)
Conduct Disorder	5.29 (2.91-9.62)*	29.9(<.001)*	9.81 (4.71-20.42)*	37.4(<.001)*	1.61 (0.90-2.89)	2.6(0.11)	2.53 (0.96-6.66)	3.5(0.06)	3.56 (1.37-9.25)*	6.8(0.009)*
ADD	1.95 (1.04-3.66)*	4.3(0.037)*	2.44 (0.86-6.92)	2.8(0.09)	1.11 (0.40-3.07)	0.0(0.84)	2.01 (0.67-6.01)	1.6(0.21)	0.68 (0.08-5.50)	0.1(0.71)
IED	4.08 (3.10-5.36)*	101.7(<.001)*	4.39 (2.81-6.86)*	42.6(<.001)*	1.26 (0.79-2.03)	0.9(0.33)	1.58 (0.76-3.29)	1.5(0.22)	1.52 (0.77-2.97)	1.5(0.22)
IV. Substance Use Disorders										
Alcohol abuse or dependence	2.79 (2.16-3.61)*	61.8(<.001)*	3.93 (2.57-6.00)*	40.2(<.001)*	1.72 (1.18-2.49)*	8.2(0.004)*	1.35 (0.86-2.12)	1.7(0.19)	1.54 (0.67-3.51)	1.0(0.31)
drug abuse or dependence	2.38 (1.45-3.93)*	11.7(<.001)*	4.37 (2.11-9.06)*	15.8(<.001)*	1.62 (0.73-3.59)	1.4(0.23)	1.50 (0.66-3.42)	0.9(0.33)	3.28 (1.15-9.40)*	4.9(0.026)*
continuous variable for # of other disorders[b]	0.78 (0.61-0.99)*	4.0(0.045)*	0.74 (0.52-1.06)	2.7(0.10)	1.04 (0.77-1.40)	0.1(0.80)	0.78 (0.51-1.19)	1.4(0.24)	0.88 (0.48-1.59)	0.2(0.67)
**Interactions with Number of Other Disorders**										
I. Anxiety Disorders										
Panic Disorder	0.97 (0.79-1.19)	0.1(0.77)	0.80 (0.59-1.09)	2.0(0.15)	0.99 (0.76-1.29)	0.0(0.93)	0.88 (0.63-1.21)	0.7(0.42)	0.63 (0.40-1.02)	3.6(0.06)
GAD	1.13 (0.92-1.40)	1.4(0.24)	0.86 (0.66-1.12)	1.3(0.26)	1.02 (0.75-1.37)	0.0(0.92)	0.93 (0.70-1.24)	0.2(0.63)	0.82 (0.50-1.36)	0.6(0.45)
Specific Phobia	0.95 (0.84-1.08)	0.6(0.44)	0.97 (0.79-1.20)	0.1(0.78)	0.95 (0.80-1.13)	0.3(0.56)	1.00 (0.79-1.28)	0.0(0.98)	0.94 (0.65-1.35)	0.1(0.72)
Social Phobia	0.87 (0.73-1.02)	2.9(0.09)	0.96 (0.79-1.17)	0.2(0.69)	1.22 (0.99-1.49)	3.7(0.06)	0.91 (0.70-1.20)	0.4(0.51)	1.09 (0.72-1.63)	0.2(0.69)
PTSD	0.79 (0.63-0.98)*	4.8(0.029)*	0.73 (0.56-0.96)*	5.0(0.025)*	0.83 (0.63-1.10)	1.6(0.20)	0.66 (0.51-0.85)*	10.3(0.001)*	0.91 (0.51-1.64)	0.1(0.76)
OCD	1.03 (0.80-1.31)	0.0(0.84)	1.09 (0.79-1.52)	0.3(0.60)	1.00 (0.71-1.40)	0.0(0.99)	1.18 (0.79-1.74)	0.6(0.42)	1.21 (0.53-2.77)	0.2(0.66)
SAD	0.96 (0.83-1.11)	0.3(0.58)	1.01 (0.83-1.22)	0.0(0.92)	1.00 (0.84-1.20)	0.0(0.99)	1.09 (0.84-1.41)	0.4(0.52)	1.21 (0.82-1.79)	0.9(0.33)
Agoraphobia	1.19 (0.99-1.43)	3.6(0.06)	1.09 (0.84-1.42)	0.5(0.50)	0.96 (0.75-1.22)	0.1(0.73)	0.81 (0.59-1.11)	1.7(0.20)	0.85 (0.53-1.37)	0.4(0.51)
II. Mood Disorders										
MDD	0.81 (0.71-0.94)*	7.9(0.005)*	0.88 (0.73-1.06)	1.8(0.18)	0.84 (0.70-1.02)	3.1(0.08)	1.08 (0.85-1.36)	0.4(0.54)	1.14 (0.73-1.79)	0.3(0.56)
Dysthymia	0.83 (0.65-1.05)	2.5(0.11)	0.89 (0.67-1.18)	0.7(0.41)	0.81 (0.64-1.02)	3.1(0.08)	1.02 (0.73-1.42)	0.0(0.91)	1.37 (0.68-2.74)	0.8(0.37)
Bipolar Disorder	0.89 (0.73-1.09)	1.3(0.26)	0.86 (0.63-1.17)	0.9(0.34)	0.66 (0.50-0.86)*	9.3(0.002)*	1.15 (0.76-1.73)	0.4(0.50)	1.31 (0.64-2.71)	0.6(0.46)
III. Impulse-Control Disorders										
ODD	0.80 (0.65-1.00)*	4.0(0.046)*	0.77 (0.58-1.04)	2.9(0.09)	0.87 (0.63-1.21)	0.7(0.41)	1.00 (0.70-1.43)	0.0(0.98)	1.21 (0.59-2.47)	0.3(0.60)
Conduct Disorder	0.79 (0.63-1.00)*	4.0(0.046)*	0.68 (0.52-0.88)*	8.2(0.004)*	0.90 (0.69-1.17)	0.6(0.43)	1.10 (0.76-1.60)	0.3(0.60)	0.38 (0.22-0.66)*	12.2(<.001)*
ADD	1.04 (0.81-1.33)	0.1(0.77)	1.02 (0.72-1.45)	0.0(0.90)	0.95 (0.68-1.33)	0.1(0.78)	0.84 (0.58-1.21)	0.8(0.36)	1.63 (0.80-3.30)	1.8(0.18)
IED	0.78 (0.67-0.91)*	10.0(0.002)*	0.78 (0.63-0.97)*	5.1(0.024)*	1.06 (0.85-1.32)	0.3(0.60)	0.89 (0.66-1.21)	0.5(0.47)	0.88 (0.60-1.29)	0.5(0.50)
IV. Substance Use Disorders										
Alcohol abuse or dependence	0.81 (0.69-0.97)*	5.6(0.017)*	0.92 (0.74-1.14)	0.6(0.45)	0.84 (0.69-1.02)	3.0(0.09)	1.03 (0.82-1.30)	0.1(0.79)	1.29 (0.80-2.07)	1.1(0.30)
drug abuse or dependence	1.14 (0.91-1.42)	1.4(0.24)	0.93 (0.69-1.26)	0.2(0.65)	1.11 (0.81-1.51)	0.4(0.51)	0.97 (0.70-1.34)	0.0(0.85)	0.57 (0.31-1.05)	3.3(0.07)
df interaction test[c]		69.9(<.001)*		62.3(<.001)*		49.6(<.001)*		18.9(0.33)		36.3(0.004)*
(N)[d]	(26959)		(26959)		(3326)		(1438)		(1888)	

Abbreviations: GAD, Generalized Anxiety Disorder; PTSD, Posttraumatic Stress Disorder; OCD, Obsessive Compulsive Disorder; SAD, Separation Anxiety Disorder; MDD, Major Depressive Disorder; ODD, Oppositional Defiant

* Significant at the .05 level, two-sided test

[a]Each column includes a separate multivariate model in survival framework, with all rows as predictors controlling for the following covariates: age, age-squared, age cohort, sex, and person-year.

[b]Number of other disorders represents the number of disorders in addition to the first one counted, so cases with 1 disorder will have 0 other disorders, cases with 2 disorders will have 1 other disorders, and so on. This is used in the multivariate model because the individual disorders are also in the model, and including a continuous number of disorder from 0,1,2,... will cause the model to overfit.

[c]Multi-df tests assess the overall interactions. This group effect test only includes all of the interactions and not all of the independent variables.

[d]Denominator sample size of the models.
