# Supplementary material for: Cross-National Analysis of the Associations among Mental Disorders and Suicidal Behavior: Findings from the WHO World Mental Health Surveys
Source: PLoS Med. 2009 Aug 11;6(8):e1000123. doi: 10.1371/journal.pmed.1000123 (PMC2717212; doi:10.1371/journal.pmed.1000123)
Supplement: Table S4 — Multivariate survival models of interactive associations between type and number of temporally primary lifetime DSM-IV/CIDI disorders in predicting the subsequent first occurrence of suicidal behaviors—developing countries. (0.02 MB PDF) [file pmed.1000123.s004.pdf]

Table S1. Prevalence of lifetime DSM-IV disorders among suicidality in developed countries

	Among total sample						Among total sample						Among Ideators						Among ideators with a lifetime plan						Among ideators without a lifetime plan					
	% with disorder among						% with disorder among						% with disorder among						% with disorder among						% with disorder among					
	Ideation			No ideation			Attempt			No attempt			Plan			No plan			Attempt			No attempt			Attempt			No attempt		
	N[2]	%[3]	SE	N[2]	%[3]	SE	N[2]	%[3]	SE	N[2]	%[3]	SE	N[2]	%[3]	SE	N[2]	%[3]	SE	N[2]	%[3]	SE	N[2]	%[3]	SE	N[2]	%[3]	SE	N[2]	%[3]	SE
I. Anxiety Disorders																														
Panic Disorder[4]	243	3.7	0.3	850	1.7	0.1	125	6.9	0.7	1101	2.1	0.1	131	5.9	0.6	240	5.8	0.4	88	7.5	0.9	77	7.8	1.1	37	6.0	1.0	174	5.0	0.5
General Anxiety Disorder[4]	454	7.5	0.4	1677	3.4	0.1	210	12.0	0.9	2349	4.4	0.1	223	11.1	0.9	629	15.9	0.7	147	12.7	1.3	178	20.4	1.7	63	10.7	1.4	494	14.5	0.7
Specific Phobia[4]	1077	17.8	0.6	3230	6.7	0.2	417	23.7	1.3	3961	7.5	0.2	451	22.5	1.1	700	17.2	0.7	284	25.7	1.6	181	20.7	1.5	133	20.1	1.9	550	16.3	0.8
Social Phobia[4]	894	15.1	0.6	1861	4.0	0.1	346	20.3	1.2	2531	4.9	0.1	404	20.9	1.2	600	15.0	0.8	245	22.8	1.5	188	21.9	1.8	101	15.9	2.0	482	14.4	0.8
Post-Traumatic Disorder[5]	489	7.7	0.4	1050	2.8	0.1	252	13.9	1.0	1556	3.6	0.1	268	11.8	0.9	458	12.0	0.7	181	14.9	1.3	169	17.8	1.7	71	12.0	1.5	337	10.4	0.7
Separation Anxiety Disorder[6]	220	4.8	0.5	519	1.8	0.1	106	7.3	0.7	718	2.3	0.1	109	6.8	1.0	199	7.1	0.7	73	8.0	1.0	59	8.2	1.5	33	6.1	1.2	140	6.3	0.9
Agoraphobia[4]	119	1.8	0.2	325	0.7	0.0	49	2.5	0.4	432	0.8	0.0	54	2.6	0.4	100	2.2	0.3	42	3.3	0.6	23	2.9	0.7	7	1.0	0.4	84	2.1	0.3
Any Anxiety Disorder[5]	2096	37.2	1.0	5850	15.3	0.3	827	49.2	1.6	7596	17.7	0.4	929	46.8	1.5	1607	44.2	1.3	574	52.4	2.0	463	54.0	2.3	253	43.3	2.6	1283	42.1	1.3
II. Mood Disorders																														
Major Depression[4]	1081	18.2	0.6	5228	11.0	0.2	466	27.0	1.3	6949	13.3	0.2	546	27.7	1.3	1536	38.4	0.9	339	30.2	1.8	470	52.2	1.9	127	21.3	1.9	1251	36.8	1.0
Dysthymia[4]	261	4.3	0.3	676	1.4	0.1	117	6.5	0.8	1024	1.9	0.1	144	6.9	0.7	321	7.9	0.5	86	7.4	1.0	101	11.6	1.3	31	5.0	1.1	247	7.0	0.5
Bipolar[4]	183	2.9	0.3	580	1.2	0.1	98	5.9	0.7	853	1.5	0.1	98	4.3	0.5	268	6.4	0.5	71	5.9	0.8	74	6.4	0.9	27	5.8	1.4	199	5.4	0.4
Any Mood[4]	1226	20.5	0.6	5797	12.2	0.2	514	29.9	1.4	7677	14.7	0.2	603	30.1	1.4	1693	42.4	0.9	370	32.6	1.9	496	54.5	2.1	144	25.1	2.1	1384	40.7	1.0
III. Impulse-Control Disorders																														
ODD[7]	182	6.5	0.7	301	1.8	0.2	99	10.5	1.1	407	2.1	0.2	92	9.5	1.4	110	6.0	0.7	66	10.5	1.4	32	9.4	2.6	33	10.5	1.7	74	4.9	0.7
Conduct[7]	154	6.0	0.7	270	1.7	0.1	81	8.9	1.0	358	2.0	0.2	74	7.4	1.3	96	6.0	1.0	51	8.2	1.4	27	7.2	2.3	30	10.3	1.8	61	4.9	1.0
ADD[7]	177	6.2	0.7	303	1.8	0.2	91	9.6	1.1	395	2.1	0.2	97	9.3	1.3	85	4.8	0.7	68	10.4	1.3	31	8.4	2.2	23	8.2	1.9	61	4.1	0.8
IED[4]	191	3.4	0.3	507	1.1	0.1	73	4.9	0.5	674	1.4	0.1	89	4.8	0.6	156	4.1	0.4	50	5.4	0.7	47	5.1	0.8	23	3.9	0.8	120	3.7	0.4
Any Impulse[7]	426	14.7	1.0	828	4.9	0.3	192	19.7	1.5	1099	5.8	0.3	195	18.1	1.7	267	14.8	1.1	128	20.0	1.9	76	17.3	2.7	64	19.2	2.3	195	13.4	1.2
IV. Substance Use Disorders																														
Alcohol Abuse or Dependence[8]	589	9.5	0.4	2776	6.2	0.2	260	14.6	1.0	3538	7.2	0.2	289	14.1	1.0	715	17.9	0.8	177	15.4	1.3	214	25.7	1.9	83	13.1	1.6	548	16.3	0.8
Drug Abuse or Dependence[9]	317	6.1	0.4	952	2.7	0.1	169	9.8	0.9	1349	3.4	0.1	189	10.2	0.8	366	10.5	0.7	128	11.4	1.1	122	16.1	1.6	41	6.9	1.3	275	9.6	0.7
Any Substance Disorder[9]	673	12.4	0.6	2551	7.6	0.2	315	18.6	1.1	3359	8.9	0.2	350	18.6	1.2	757	22.4	0.9	218	20.0	1.5	229	29.8	2.1	97	16.2	1.9	579	20.8	1.0
Any Disorder[5]	2865	51.8	1.1	10254	27.3	0.4	1096	65.7	1.7	12886	30.7	0.5	1237	63.4	1.7	2409	68.2	1.2	750	68.6	2.0	670	79.3	2.0	346	60.5	2.8	1962	66.3	1.3
V. Number of Disorders																														
Exactly 1[5]	1212	22.2	0.8	5548	16.1	0.3	366	22.0	1.3	6404	16.8	0.3	423	21.8	1.3	785	23.2	0.9	239	21.7	1.6	180	23.1	2.1	127	22.4	2.1	676	24.0	1.0
Exactly 2[5]	711	12.6	0.6	2534	6.3	0.2	258	15.2	1.1	3208	7.2	0.2	307	14.9	1.0	615	17.3	0.8	172	15.3	1.4	153	16.8	1.5	86	14.8	1.8	521	17.3	0.9
Exactly 3[5]	426	7.7	0.4	1108	2.5	0.1	200	12.1	1.0	1576	3.3	0.1	201	10.0	0.9	414	11.3	0.7	142	13.1	1.4	129	14.4	1.6	58	10.2	1.5	339	11.1	0.7
Exactly 4[5]	236	4.4	0.4	571	1.3	0.1	114	6.9	0.8	850	1.8	0.1	130	7.7	0.9	264	7.6	0.6	80	7.4	0.9	80	10.4	1.8	34	5.9	1.4	199	6.7	0.6
Exactly 5[5]	141	2.5	0.2	250	0.6	0.1	66	3.7	0.5	408	0.9	0.1	69	3.0	0.4	139	3.8	0.5	46	3.8	0.6	54	5.4	0.9	20	3.7	0.9	104	3.5	0.4
6 or more disorders[5]	139	2.5	0.3	243	0.5	0.0	92	6.0	0.8	440	0.9	0.1	107	6.0	0.8	192	5.0	0.4	71	7.4	1.1	74	9.1	1.4	21	3.5	0.9	123	3.8	0.4
(N)[1]		(5869)			(45902)			(1700)			(50071)			(1969)			(3900)			(1110)			(859)			(590)			(3310)	

[1] Number of cases with the outcome variable

[2] Numerator N. Number of cases with the LT disorder among cases with the outcome variable. Columns with "Yes" in the headers represents cases with the dx among those with the outcome, and columns with "No" represents among those without the outcome. Part II disorders assessed in part II sample, and disorders with age restrictions restricted to appropriate age range

[3] % represents the percentage of people with the DSM-IV disorder among the cases with the outcome variable indicated in the column header. For example: the first cell is the % of those with Panic disorders among those with attempts

[4] assessed in part I sample

[5] assessed in part II sample

[6] assessed in part II sample. Restricted to age groups 18-44 for Esemed data (Beligum, France, Germany, Italy, Netherlands, Spain), but not other countries.

[7] assessed in part II sample and restricted to age groups 18-44.

[8] assessed in part II sample for US and Japan, while in part I sample for the remaining countries. A weight variable is created that takes the part II weight values for data in US and Japan and part I weight for others.

[9] assessed in part II sample for all data except New Zealand, where part I weight is used. A weight variable is created that takes the part I weight values for New Zealand and part II for other countries.
